# KLF2 Promotes Ellagic Acid-Mediated Osteogenic Differentiation of Dental Pulp-Derived Stem Cells via Autophagy and Mitochondrial Regulation

**DOI:** 10.54457/dr.202504006

**Published:** 2026-02-11

**Authors:** Prathyusha Naidu, Md Sariful Islam Howlader, Surajit Hansda, Manjusri Das, Hiranmoy Das

**Affiliations:** 1 Department of Pharmaceutical Sciences, Jerry H. Hodge School of Pharmacy, Texas Tech University Health Sciences Center, Amarillo, Texas 79106, USA

**Keywords:** DPSC, Transcription factor KLF2, Osteogenic differentiation, GGTI298, GGPP

## Abstract

**Backgrounds:**

Krüppel-like factor 2 (KLF2), a zinc-finger transcription factor of the Kruppel-like factor family, plays a crucial role in regulating various cellular processes, including differentiation, autophagy, and metabolism. However, it is not clear whether it has any role in the ellagic acid (EA)-mediated osteoblastic differentiation of dental pulp-derived stem cells (DPSCs). To investigate any regulatory role of KLF2 during EA-induced osteoblastic differentiation of DPSC, we have evaluated the path-ways emphasizing autophagy, mitophagy, and mitochondrial bioenergetics.

**Methods:**

We used induction and reduction of KLF2 approaches using chemical compounds, such as geranylgeranyl transferase inhibitor 298 (GGTI298), a known inducer of KLF2, and geranylgeranyl pyrophosphate (GGPP), a known inhibitor of KLF2. The key osteogenic, autophagy, and mitophagy markers were assessed via RT-qPCR and Western blotting, intracellular and mitochondrial ROS, along with mitochondrial membrane potential using high-resolution confocal microscopy, and cellular bioenergetics using Seahorse XF methods.

**Results:**

We found that EA alone significantly upregulated osteogenic markers, along with enhanced expression of autophagy and mitophagy-related molecules, and mitochondrial biogenetics. However, when we induced the KLF2, it amplified the expression of these markers and improved mitochondrial bioenergetics, suggesting a distinct relation between EA and KLF2. On the other hand, inhibition of KLF2 led to a significant downregulation of these markers.

**Conclusion:**

This supports the notion that KLF2 is a pivotal transcriptional regulator involved in mediating the pro-osteogenic effects of EA. It’s activation enhanced autophagy, and improved mitochondrial bioenergetics, thereby facilitating EA-induced osteogenic differentiation of DPSCs. These findings refine our understanding of molecular mechanisms underlying polyphenol-mediated osteogenesis and show the pivotal role of KLF2 for regeneration via osteogenic differentiation of DPSCs.

## Graphical Abstract

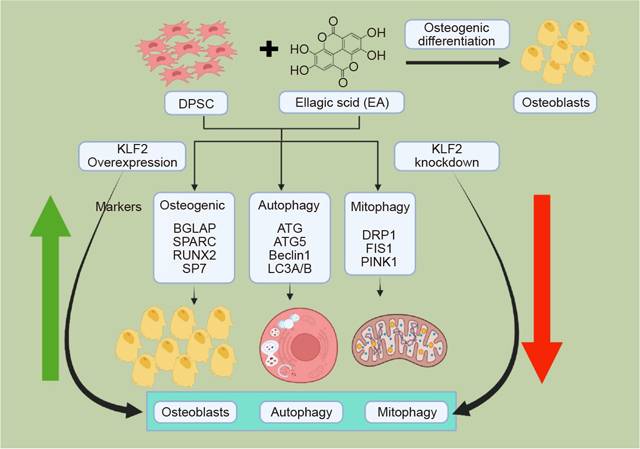

## Introduction

Ellagic acid (EA), C_15_H_10_O_7,_ is a polyphenolic compound found predominantly in plants. It is mainly present in fruits and nuts, with the highest levels reported in berries, pomegranates, and certain nuts such as walnuts. EA exhibits antioxidant, anti-inflammatory, and antimicrobial properties, contributing to human health^[1]^. When combined with hydroxyapatite, EA has been shown to stimulate bone growth in models of bone defects, indicating a synergistic effect that promotes bone regeneration^[2]^. Additionally, EA has been studied for its potential to prevent bone loss associated with postmenopausal osteoporosis^[3]^. In ovariectomized mice, a model for postmenopausal osteoporosis, EA suppressed bone resorption and enhanced osteoblast differentiation. This activity was linked to bone health by regulating the balance between osteoclasts and osteoblasts^[4,5]^. However, the upstream transcriptional regulators through which EA mediates these effects are not fully understood.

Bone regeneration is a complex process that involves the development of osteoblast differentiation, osteoclast activity, formation of the extracellular matrix to maintain bone homeostasis, and repair in case of injury^[4,6]^. On the other hand, it is essential to comprehend the molecular and cellular processes that govern bone physiology and pathology to create treatments for any bone disease^[7]^. Bone has a special characteristic that makes it different from many other tissues; it possesses a capability to fully regenerate rather than form a scar following an injury^[8]^.

Upon an injury after the initial inflammatory phase, mesenchymal stem cells (MSCs) and blood vessels are activated at the site of injury and start to regenerate into osteoblasts or chondrocytes^[9]^. One of the most commonly used cells for regenerative therapies is the stem cells, which are the MSCs, because they can be easily isolated, with multipotency and differentiability to the bone-forming cells^[10]^. It is possible to culture MSCs and direct them to undergo osteogenic differentiation in a controlled environment, for instance, under the administration of natural compounds, BMPs, dexamethasone, ascorbic acid, and β-glycerophosphate^[11]^. Among various MSCs, Human DPSCs are promising for regenerative medicine, as they are extracted from the soft tissue within teeth. These MSCs are notable for their multipotent differentiation and regenerative therapeutic potential^[12,13]^. Moreover, the development of these MSCs into various lineages, such as osteoblasts, adipocytes, is strictly controlled by transcriptional regulators like Kruppel-like factor 2 (KLF2)^[14]^. These transcription factors are of great importance in directing the lineage commitment of MSCs towards the osteogenic differentiation while inhibiting them towards adipogenesis^[15]^.

Bone growth and homeostasis are controlled by the equilibrium of the osteocytes (the mature cells of the bone in the matrix), the osteoclasts (cells that resorb the bones), and the osteoblasts (bone-forming cells). Bone repair, generation, and formation are all founded on the process of osteogenesis, where the MSCs are converted to osteoblasts. Imbalances in this equilibrium may give rise to skeletal system diseases like osteoporosis, which causes the bones to lose their mass and have a greater chance of fracturing^[16]^. KLF2 plays a role in bone formation, as well as its maintenance through the regulation of osteoblast differentiation, osteoclastogenesis, and the formation of bone matrix through different mechanisms^[17]^. Among the pathways through which KLF2 regulates the homeostasis of bones, the regulation of the Wnt signaling pathway, which is vital for osteoblast differentiation and the formation of bones. KLF2 can resultantly induce the expression of RUNX2 (Runt-related transcription factor 2), a singledout transcription factor, which can be improved toward enhanced commitment for MSCs^[18]^.

KLF2 is a transcription factor from the KLF family that plays important roles in many biological processes such as development, differentiation, homeostasis, and disease pathogenesis. Initially identified for its role in endothelial function, KLF2 is also expressed in several other cell types, including MSCs, osteoblasts, and osteoclasts, which are involved in bone development and maintenance^[19]^. KLF2 is essential for maintaining the integrity of various tissues and organs by regulating gene expression in response to environmental stimuli. Its activity is controlled through multiple mechanisms, such as interactions with cofactors, phosphorylation, and regulation of expression levels depending on environmental signals^[20]^. Understanding KLF2’s function in bone biology could reveal the molecular basis of bone diseases, such as osteoporosis, and may lead to new therapeutic strategies for enhancing bone repair^[21]^. Bone growth is closely linked to angiogenesis, the formation of new blood vessels, and KLF2 has been shown to regulate genes involved in both angiogenesis and osteogenesis^[22]^. By regulating endothelial cell functions and contributing to vascular stability, KLF2 facilitates oxygen and nutrient delivery to growing and regenerating bone tissue^[17]^. Besides its role in osteoblasts, KLF2 also affects osteoclasts, which resorb bone material through processes regulated by signals such as Receptor activator of nuclear factor kappa-B ligand (RANKL)^[23]^. KLF2 influences osteoclastogenesis, and by modulating autophagy, it regulates osteoclast differentiation and activity, helping maintain the balance between bone resorption and formation^[24]^. KLF2’s ability to regulate both osteoclasts and osteoblasts places it at a central point in skeletal homeostasis regulation. Additionally, KLF2’s role in osteogenesis has been extensively studied. Modulating KLF2 expression either through induction or inhibition affects osteoblast differentiation and bone matrix development, highlighting its importance in bone formation^[25]^. Despite these insights, there are no studies that have examined whether EA could modulate KLF2 signaling to direct osteogenic differentiation of DPSCs, nor whether KLF2 serves only as the mechanistic link between EA’s cytoprotective effects and the metabolic remodeling that is required for osteogenesis. This knowledge gap is important to understand as the given emerging evidence indicates that KLF2 regulates autophagy-related gene networks and mitochondrial quality control, processes that are increasingly recognized as determinants of osteogenic differentiation^[24]^.

Among many intracellular processes during osteoblast differentiation, two of the most important are autophagy and mitophagy, along with maintaining proper mitochondrial dynamics. Autophagy functions as a quality-control mechanism that degrades and recycles damaged organelles and proteins. This helps preserve homeostasis during both basal and stressful conditions. Thus, differentiation is a tightly regulated process that requires high energy and active organelles. During this process, autophagy supports metabolic remodeling essential for lineage commitment by maintaining the availability of precursors needed for bioenergetics and biosynthesis^[26,27]^. Studies have shown that inhibiting autophagy hampers osteoblast differentiation and mineralization, underscoring its vital role in bone formation^[28]^. Similarly, mitophagy helps eliminate damaged mitochondria, preventing reactive oxygen species (ROS) buildup and facilitating the proper shift from glycolysis to oxidative phosphorylation, which is critical for osteoblast maturation^[29,30]^. Conversely, disruption of these processes can cause dysregulation in differentiation, leading to bone loss. Mitochondrial dynamics, comprising fission, fusion, and biogenesis, are essential for the bioenergetic reprogramming of stem cells during osteogenesis. An increase in mitochondrial activity provides the ATP necessary for matrix production and mineralization.

Although KLF2 has been implicated in stem-cell osteogenesis by regulating mitophagy, and EA is known for its antioxidative and osteogenic actions, those properties have been independently reported. But there are no prior studies that have examined whether EA and KLF2 have any relationship that aids the osteogenesis process. So, our study aims to explore how the osteogenic differentiation of DPSC mediated by EA influences the level of KLF2 and how changes in KLF2 expression levels impact the levels of autophagy, mitophagy, and mitochondrial functions. We emphasized establishing that KLF2 is a target molecule for the EA-mediated osteoblast differentiation of DPSCs and showed that modulating KLF2 levels elevated osteogenesis.

## Materials and Methods

### Chemicals and reagents

Various reagents and materials used in this study were sourced from well-established suppliers. α-Modified Essential Medium (α-MEM, #M8042–500 ML) and Dulbecco’s Modified Eagle Medium (DMEM, #D6046–500 ML), GGTI-298 (#G5169), geranylgeranyl pyrophosphate (GGPP, #G6025), Ponceau S (#P7170), bovine serum albumin (BSA, #A9418–100), DCFDA dye (#D6883), and ellagic acid (#E2250–1G) were purchased from Sigma-Aldrich (St. Louis, MO, USA). Fetal bovine serum (FBS, #PS-FB3) was obtained from Peak Serum (Wellington, CO, USA). Additional cell culture reagents, including penicillin-streptomycin (#10378–016), Antibiotic-Antimycotic solution (#15240062), L-glutamine (#25030081), trypsin (#25200–056), Cell Dissociation Buffer (#13151014), and phosphate-buffered saline (PBS, #70013–032) were procured from Fisher Scientific, Gibco (Waltham, MA, USA).

RNA extraction and fluorescent staining reagents such as TRIzol reagent (#15596018), JC-1 dye (#T3168), MitoSOX Red (#M36008), and antifade mounting medium (#P36931) were purchased from Invitrogen, a division of Fisher Scientific (Waltham, MA, USA). The cDNA synthesis kit (#4387406) and SYBR Green PCR kit (#4309155) were obtained from Applied Biosystems (Waltham, MA, USA). For protein analysis, Pierce RIPA lysis buffer (#89901) and SuperSignal West Pico PLUS chemiluminescent substrate (#34578) were purchased from Thermo Fisher Scientific (Waltham, MA, USA).

Materials required for SDS-PAGE and Western blotting, including TEMED (#161–0800), Bradford reagent (#500–0006), nitrocellulose membranes (0.45 μm, #1620115), and filter paper (#1650962), were obtained from Bio-Rad Laboratories (Hercules, CA, USA). Protogel (#EC-890) was sourced from National Diagnostics (Atlanta, GA, USA). Additional chemicals such as SDS (#BP1311–1), DMSO (#BP231–100), and methanol (#A412P-4) were acquired from Fisher Scientific (Hampton, NH, USA). Electrophoresis and blotting buffers, including separating buffer (#BP-90), stacking buffer (#BP-95), running buffer (#BP-150), transfer buffer (#BP-190), and TBST (#IBB-180) were purchased from Boston Bioproducts (Milford, MA, USA). Nonfat dry milk (#M0841) was obtained from LabScientific (Highlands, NJ, USA), and paraformaldehyde (#sc-281692) was procured from Santa Cruz Biotechnology (Dallas, TX, USA). A molecular weight marker (#RPN800E) was obtained from Sigma, St. Louis, MO, USA.

Hanks’ Balanced Salt Solution (#HBSS, 21–020-CV) was purchased from Mediatech Inc. (Manassas, VA, USA), while DEPC-treated water (#AM9922) was obtained from Ambion, a division of Thermo Fisher Scientific. Seahorse XF assay reagents including the Cell Mito Stress Test Kit (#103035–100), Glycolysis Stress Test Kit (#103020–100), XF Base Medium (#102353–100), Glucose Solution (#103577–100), Pyruvate Solution (#103578–100), Glutamine Solution (#103579–100), and XF Calibrant (#100840–000) were all obtained from Agilent Technologies (Santa Clara, CA, USA).

### Isolation and culture of human DPSCs

Human DPSCs were isolated and expanded following a previously published protocol^[22]^. Third molars were collected from a healthy adolescent donor under approved Institutional Review Board guidelines and with informed consent. The extracted teeth were rinsed thoroughly three times with PBS containing 1% antibiotic-antimycotic solution to minimize contamination. Dental pulp tissue was extracted by sectioning the teeth, finely minced into ~ 1 mm fragments, and plated onto 60 mm culture dishes. The cells were grown in α-MEM supplemented with 20% FBS and 1% antibiotic-antimycotic solution at 37 °C in a humidified atmosphere with 5% CO_2_. The culture medium was replaced every three days with new medium. Once cells migrated from the pulp tissue and reached confluency, they were detached using a cell dissociation buffer and subcultured at passage 1. Experimental procedures were carried out using cells between passages 2 and 7.

### Osteogenic differentiation of DPSCs

Osteogenic differentiation of DPSCs were performed following the previously published protocol^[31]^. DPSCs were cultivated in 10 cm culture dishes, employing αMEM medium that contains 20% FBS, 1% antibiotic-antimycotic solution, and 1% α-glutamine, to support their growth and proliferation. The cells were grown until they reached confluency at 37 °C in an incubator with 5% CO_2_. After attaining at least 80% confluency, the cultured DPSCs were trypsinized and then plated into a 12-well plate and grown until confluence for osteogenic differentiation with DMEM plus 10% FBS, 1% antibiotic-antimycotic solution, and 1% α-glutamine (osteogenic differentiation basic media). The control group received osteogenic differentiation basic media containing DMSO (the vehicle), whereas the experimental groups were treated with DMEM media containing a total concentration of 5 μM of EA dissolved in DMSO. Subsequently, the samples were then incubated at 37 °C with 5% CO_2_ for seven days. The media was replaced every third day.

### Induction and reduction of KLF2

To induce KLF2 expression, human DPSCs were grown until they reached confluency at 37 °C in an incubator with 5% CO_2_. After attaining at least 80% confluency, the media was replaced with DMEM basic media containing 2.5 μM GGTI298. Then, the medium was replaced with DMEM basic media containing the same concentration of GGTI298 and 5 μM EA throughout the differentiation process for 7 days. Similarly, for the reduction of KLF2 expression, the cells were cultured with 10 μM GGPP in DMEM after 24 h, the medium was replaced with DMEM basic media containing the same concentration of GGPP and 5 μM EA for 7 days^[18]^.

### Quantitative RT-PCR analysis

DPSCs were exposed to EA along with GGTI298 or GGPP for 7 days, then the cells underwent total RNA extraction using the TRIzol method and performed RT-qPCR according to our previous protocol^[32]^. Briefly, complementary DNA (cDNA) was generated from the extracted mRNA utilizing a cDNA synthesis kit. The cDNA produced was used as a template for quantitative PCR (qPCR), performed with SYBR Green chemistry on a Bio-Rad CFX96 Real-Time System^[32]^. Gene-specific primers targeting SPARC (osteonectin), BGLAP (osteocalcin), SPP1 (osteopontin), SP7 (osterix), and the housekeeping gene β-Actin were obtained from Integrated DNA Technologies. The sequences of the primers were provided in Supplementary Table S1. Quantification cycle (Cq) values were analyzed using the ΔΔCt method, with β-Actin as the internal control gene, and we assessed relative gene expression levels.

### Protein analysis

DPSCs were maintained under standard culture conditions, while the treatment groups were exposed to a total concentration of 5 μM EA along with KLF2 inducer or KLF2 inhibitor in DMEM for 7 days. At the end of the treatment period, whole-cell lysates were collected, and total proteins were estimated using the Bradford assay and performed western blotting analysis according to our previous protocol^[13]^. Briefly, an equal quantity of total protein (20 μg) was denatured, loaded onto polyacrylamide gels, and resolved by SDS-PAGE. A molecular weight protein ladder was loaded as a marker for reference. Following electrophoresis, proteins were transferred onto 0.45 μm nitrocellulose membranes at 4 °C. Membranes were incubated with a blocking solution containing 5% nonfat dry milk for one hour at room temperature in Tris-buffered saline with Tween-20 (TBST) to prevent nonspecific binding. After blocking, membranes were rinsed with TBST and incubated overnight at 4 °C with the primary antibodies specific for SPARC, BGLAP, KLF2, SP7, RUNX2, or GAPDH. The antibodies used and their dilutions are provided in the Supplementary Table S2. Every primary antibody was diluted to a ratio of 1:1000 in TBST, supplemented with 5% BSA.

Following the incubation period with the primary antibody, the membranes were washed at least three times and then treated with HRP-conjugated secondary antibodies diluted 1:3000 in 5% milk in TBST for 2 hours at room temperature. After final washes, protein bands were visualized using a chemiluminescent detection reagent, and films were developed using a film exposure machine. The band intensities were assessed utilizing ImageJ software from the NIH, and expression levels were normalized to GAPDH.

### Intracellular ROS assessment

To measure the ROS levels within cells, the fluorescent probe DCFDA (2’,7’-dichlorodihydrofluorescein diacetate) was employed. This compound is non-fluorescent until it is oxidized by ROS inside the cell, at which point it becomes fluorescent. For the experiment, DPSCs (8 × 10^4^ cells) were seeded on the sterile cover glasses in six-well plates and allowed to attach overnight. The next day, the culture medium was replaced with fresh osteogenic differentiation medium containing vehicle, EA alone, or EA along with GGTI298 or GGPP, and cells were incubated for 7 days. Every 3^rd^ day medium was replaced with appropriate fresh medium. Cells were then washed with 1 × PBS and incubated with a 5 μM solution of DCFDA diluted in 1 × PBS for 20 minutes at 37 °C. Post-incubation, cells were rinsed again with PBS at least 3 times, and fluorescence images were captured using a Leica Stellaris 8 Falcon STED confocal microscope. The fluorescence was measured at an excitation wavelength of 495 nm and an emission wavelength of 529 nm.

### Mitochondrial ROS measurement

To measure ROS produced by mitochondria, MitoSOX^™^ Red fluorescent dye was utilized. This dye is very specific to mitochondria and selectively reacts with mitochondrial superoxide, emitting red fluorescence when oxidized. For the experiment, DPSCs were transferred to each well at a density of 8 × 10^4^ on the sterile coverslips in six-well plates and incubated overnight. The following day, the culture medium was replaced with osteogenic differentiation medium containing vehicle, EA alone, or EA along with GGTI298 or GGPP, and the cells were cultured for seven days. Following the incubation period, the cells were rinsed with 1 × PBS and treated with a 5 μM solution of MitoSOX Red prepared in Hanks’ Balanced Salt Solution (HBSS). The cells were incubated with the dye for 15 minutes at 37 °C. Subsequently, they were washed with 1 × PBS for at least 3 times, and fluorescence imaging was performed using a Leica Stellaris 8 Falcon STED confocal microscope, with excitation at 510 nm and emission measured at 588 nm.

### Assessment of mitochondrial membrane potential

To measure the mitochondrial membrane potential (Δψm), the 5,5′,6,6′-tetrachloro-1,1′,3,3′-tetraethylbenzimidazolocarbocyanine iodide (JC-1) fluorescent dye was used. JC-1 is a lipophilic cationic dye that accumulates in mitochondria in a potential-dependent manner. In its monomeric form, JC-1 produces green fluorescence (~ 525 nm), whereas, at elevated mitochondrial membrane potential, JC-1 aggregates to form J-aggregates that produce red fluorescence (~ 590 nm). Thus, healthy mitochondria with intact membrane potential appear as red-stained aggregates, whereas cells with diminished Δψm predominantly produce green fluorescence.

To assess the Δψm assessment, DPSC at a density of (8 × 10^4^) were seeded onto glass coverslips and allowed to adhere overnight. The culture medium was replaced with osteogenic differentiation medium containing vehicle, EA alone, or EA along with GGTI298 or GGPP, and the cells were cultured for seven days. After the incubation period, cells were incubated with freshly prepared 5 μM concentration of JC-1 dye for 15 minutes at 37 °C in the dark. Following incubation, cells were washed with 1 × PBS at least 3 times. Fluorescence imaging was performed using a Leica Stellaris 8 Falcon STED confocal microscope. Quantitative analysis of Δψm was performed using LAS X image analysis software. The mitochondrial membrane potential was expressed as the ratio of mean fluorescence intensity of JC-1 aggregates (red, polarized mitochondria) to JC-1 monomers (green, depolarized mitochondria).

### Cellular energy metabolism assessment

To evaluate mitochondrial function and glycolytic activity during the osteogenic differentiation of DPSCs, we utilized an XF24 Extracellular Flux Analyzer. This instrument measures the oxygen consumption rate (OCR) as an indicator of mitochondrial respiration, and the extracellular acidification rate (ECAR) as a readout of glycolysis. Differentiated DPSCs were non-enzymatically detached and reseeded equally to ensure normalization of cell number at a density of 5 × 10^4^ cells per well into XF24 cell culture microplates, depending on the experimental condition. Cells were grown for 24 hours before conducting the assay. Meanwhile, the sensor cartridge was hydrated overnight in Seahorse calibrant solution in a non-CO_2_ incubator at 37 °C. Before the assay, the cultures were washed twice with Seahorse XF base medium, supplemented with pyruvate, glutamine, and glucose to support cellular metabolism. Each well received 500 μL of the supplemented medium, and plates were incubated for 1 hour at 37 °C in a non-CO_2_ environment. Subsequently, the hydrated sensor cartridge was loaded with metabolic inhibitors: oligomycin (1.5 μM) to inhibit ATP synthase, FCCP (1 μM) as a mitochondrial uncoupler, and rotenone plus antimycin A (0.5 μM each) to block electron transport at complex III. Instrument calibration was performed using the Seahorse Wave software version 2.6.1. After calibration, the test plate was inserted, and OCR measurements were obtained to assess parameters such as ATP-linked respiration, maximal respiration, and spare respiratory capacity. All the parameters were automatically corrected for non-mitochondrial oxygen consumption and background signal using the Seahorse Wave software. For glycolytic function, a glycolysis stress test was performed by sequentially injecting glucose (1 mM), oligomycin (1 μM), and 2-deoxyglucose (10 mM). ECAR values were analyzed using the same Seahorse Wave software 2.6.1.

### Statistical analysis

Each experiment was performed at least three times, and each data point was measured in triplicate. Data are presented as mean ± SEM from three independent experiments. Statistical significance was determined using one-way ANOVA followed by Tukey’s post hoc test, with *P* ≤ 0.05 considered significant.

## Results

### Effect of KLF2 on osteogenic marker expressions

**Fig. 1.**
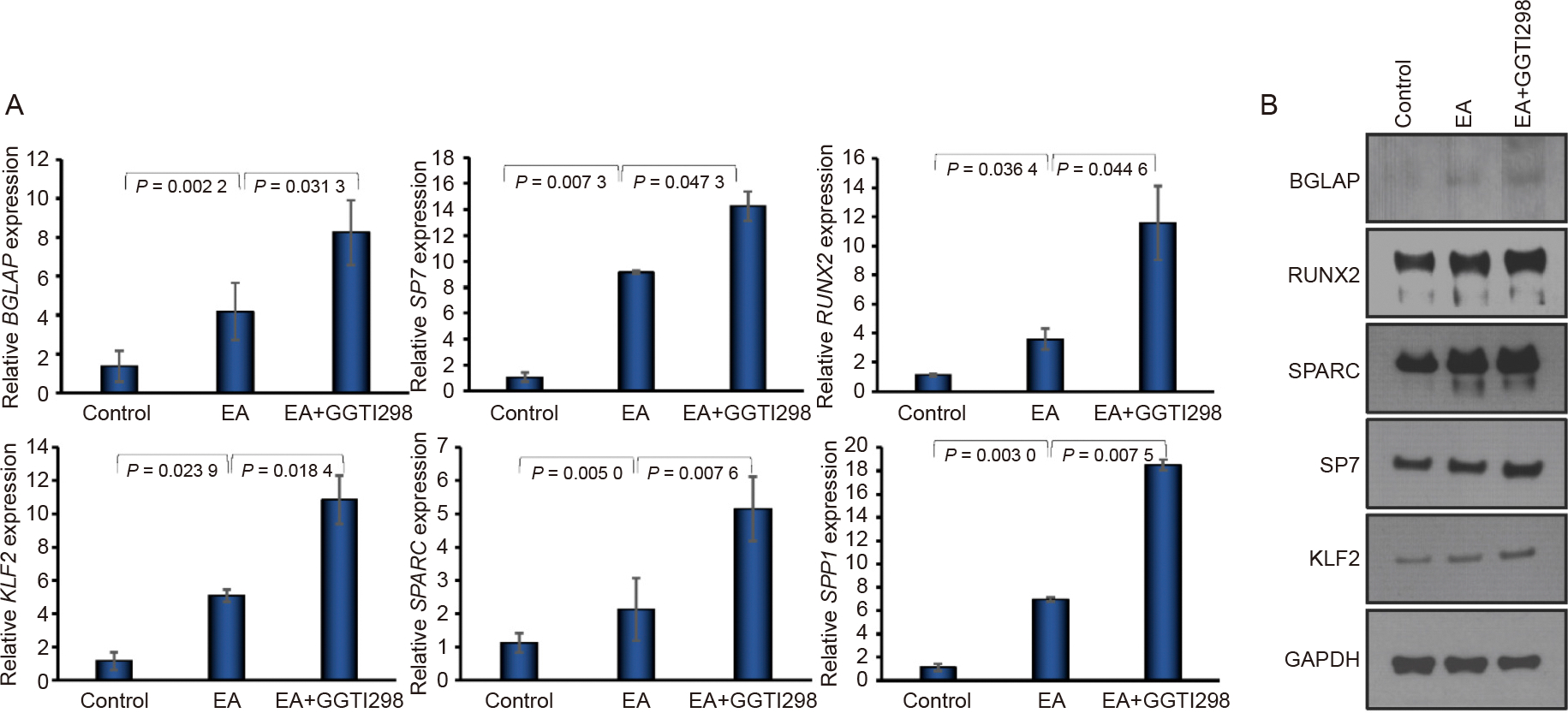
Induction of KLF2 enhanced osteogenic marker expressions. A. The mRNA expression level of BGLAP, SP7, RUNX2, KLF2, SPARC, and SPP1 was determined by qRT-PCR at 7 days of differentiation of DPSC after the addition of GGTI298 to the cells. B. Western blot images of BGLAP, RUNX2, SPARC, SP7, and KLF2 at 7 days of osteoblastic differentiation using similar experimental conditions. GAPDH was used as an internal loading control. Comparisons were conducted between control and EA and between EA and GGTI298 which were presented as fold change and shown as mean ± SEM from three independent experiments. Statistical significance was determined using one-way ANOVA followed by Tukey’s post hoc test and the exact p-values are shown on the graph.

To clarify the regulatory role of KLF2 in the osteogenic differentiation of DPSCs, we stimulated DPSCs with GGTI298 and cultured them in osteogenic differentiation medium for 7 days. Our RT-qPCR analysis demonstrated that DPSCs treated with GGTI298 showed a significantly higher expression of KLF2 compared to the control or EA-added DPSCs. Similarly, the mRNA levels of several osteoblast-specific markers, including BGLAP (Osteocalcin), SP7 (Osterix), RUNX2, SPARC (Osteonectin), and SPP1 (Osteopontin), were markedly increased after the addition of GGTI298 to the DPSCs (Fig. 1A). Similarly, western blot analysis confirmed the increased expression of several osteogenic markers, such as BGLAP, RUNX2, SP7, and SPARC, along with KLF2, when GGTI298 was added to the DPSCs compared to control or EA-added DPSCs (Fig. 1B and Supplementary Fig S1), indicating that induction of KLF2 accelerated osteogenic differentiation-related molecules at the mRNA and protein levels.

### Effect of KLF2 on autophagy/mitophagy marker expressions

**Fig. 2.**
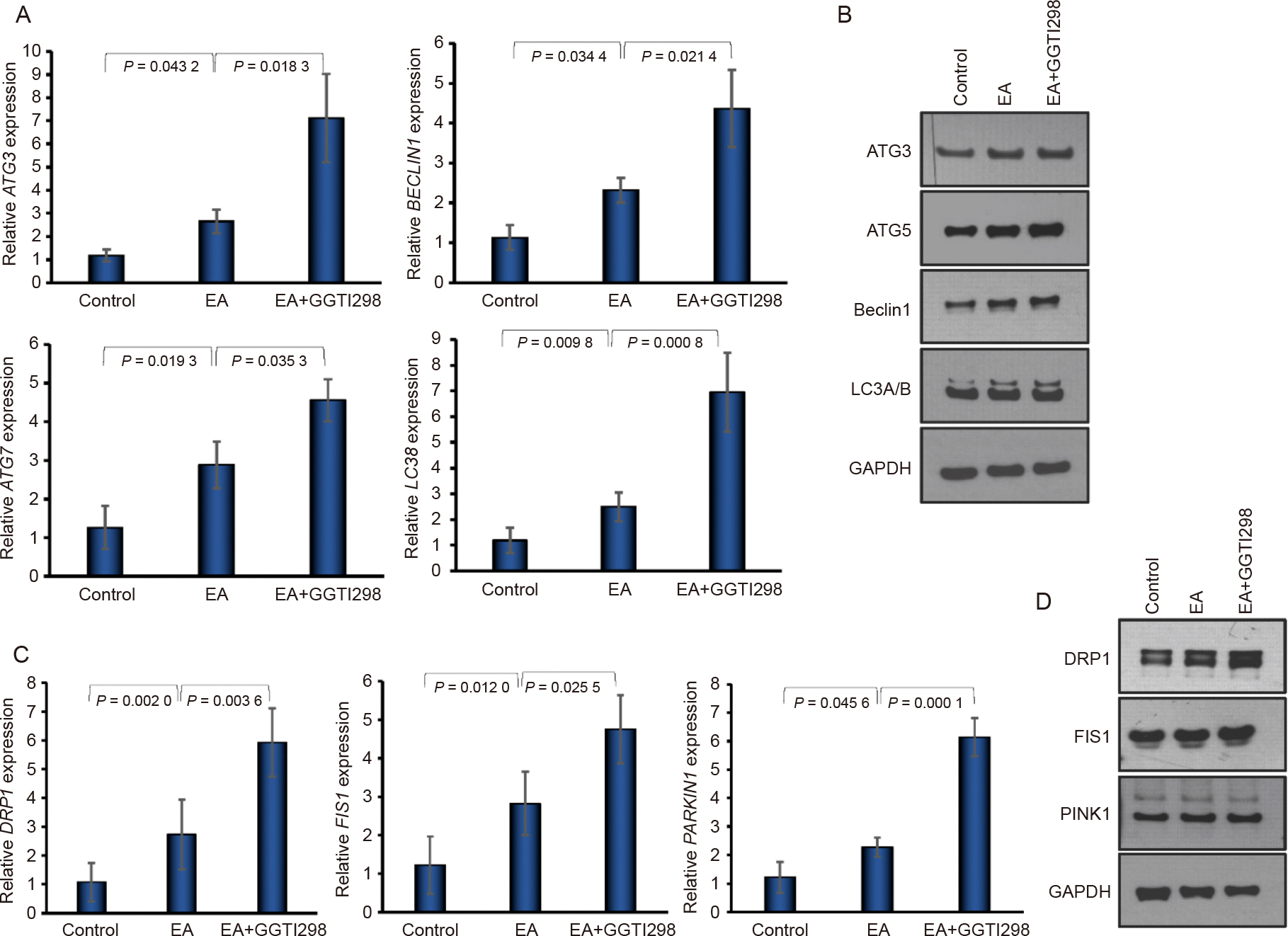
Induction of KLF2 enhanced autophagy/mitophagy marker expressions. A. Graphically shown are the qRT-PCR values of autophagy-associated markers BECN1, ATG3, ATG7, and LC3B at 7 days of differentiation of DPSC after the addition of GGTI298 to the cells. B. Images of western blot autophagy molecules ATG3, ATG5, Beclin1, and LC3A/B were shown after 7 days of osteoblastic differentiation, keeping GAPDH as an internal loading control. C. Graphically shown are the qRT-PCR values of mitophagy-associated markers PARKIN, DRP1, and FIS1 at 7 days of differentiation of DPSCs after the addition of GGTI298 to the cells. D. Western blot images of DRP1, FIS1, and PINK1 molecules were shown after 7 days of osteoblastic differentiation, keeping GAPDH as an internal loading control. Comparisons were conducted between control and EA and between EA and GGTI298 which were presented as fold change and shown as mean ± SEM from three independent experiments. Statistical significance was determined using one-way ANOVA followed by Tukey’s post hoc test and the exact p-values are shown on the graph.

To study the induction of KLF2 in the modulation of autophagyrelated markers, we evaluated the expression of key markers at both the gene and protein levels. RT-qPCR revealed a significant increase in the levels of autophagy-related genes, such as ATG3, ATG7, LC3B, and BECN1 after the addition of GGTI298 to the DPSCs compared to the control and EA-treated cells (Fig. 2A). In addition, western blot analysis supported these findings, showing elevated protein levels of autophagy markers ATG3, ATG5, Beclin-1, and LC3A/B, suggesting that induction of KLF2 might induce autophagy to protect cells (Fig. 2B). Next, we examined whether KLF2 also influences mitophagy, the selective clearance of dysfunctional mitochondria, which is crucial during stem cell differentiation and energy remodeling. Induction of KLF2 led to an elevated level of mRNA of mitophagy-related genes such as DRP1, FIS1, and PARKIN compared to control and EA-treated cells (Fig. 2C). Similarly, at the protein level, a significant increase in signal intensity for DRP1, FIS1, and PINK1 was observed in KLF2-induced DPSCs (Fig. 2D and Supplementary Fig. S2).

### Effect of KLF2 on the production of intracellular and mitochondrial ROS

**Fig. 3.**
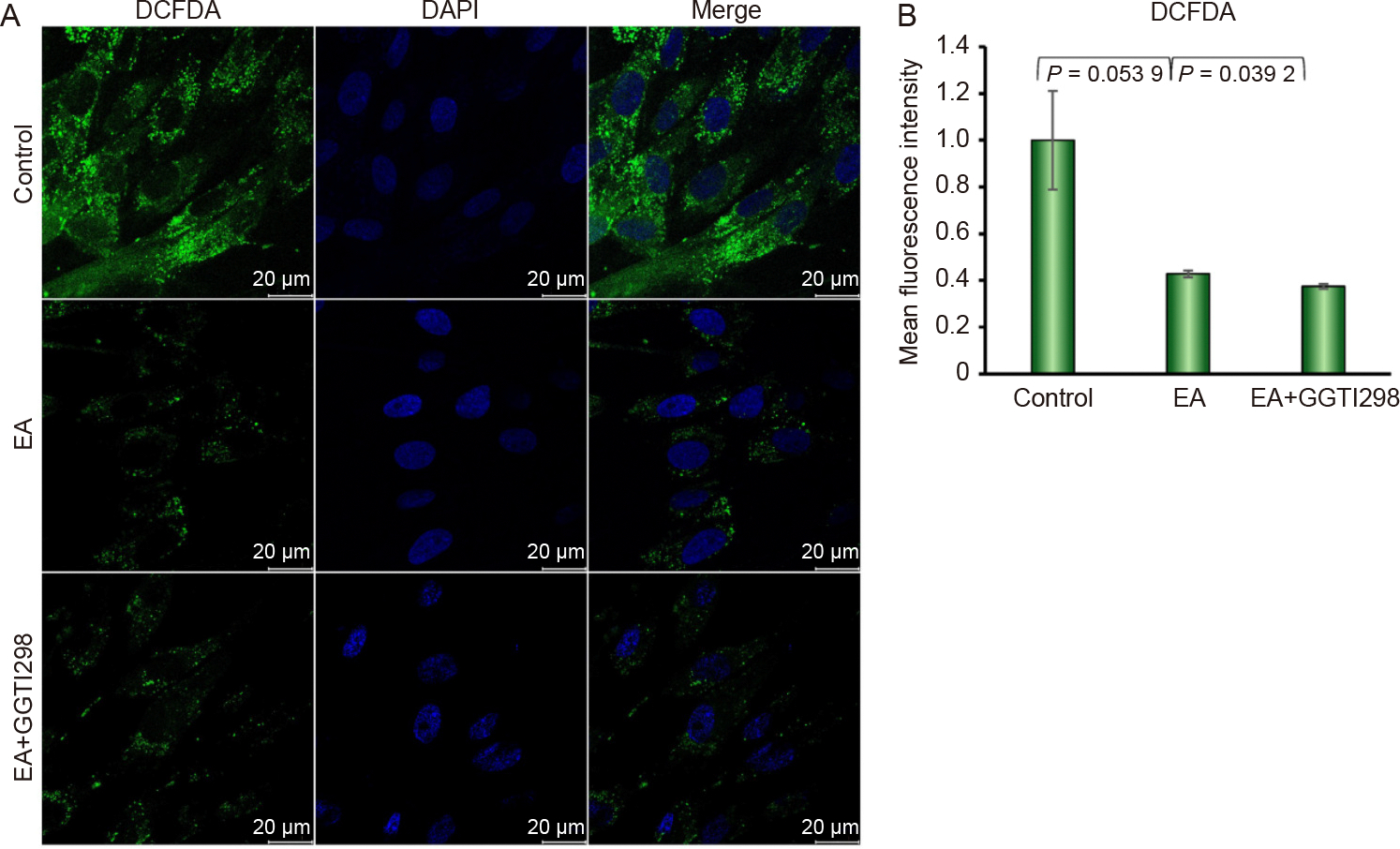

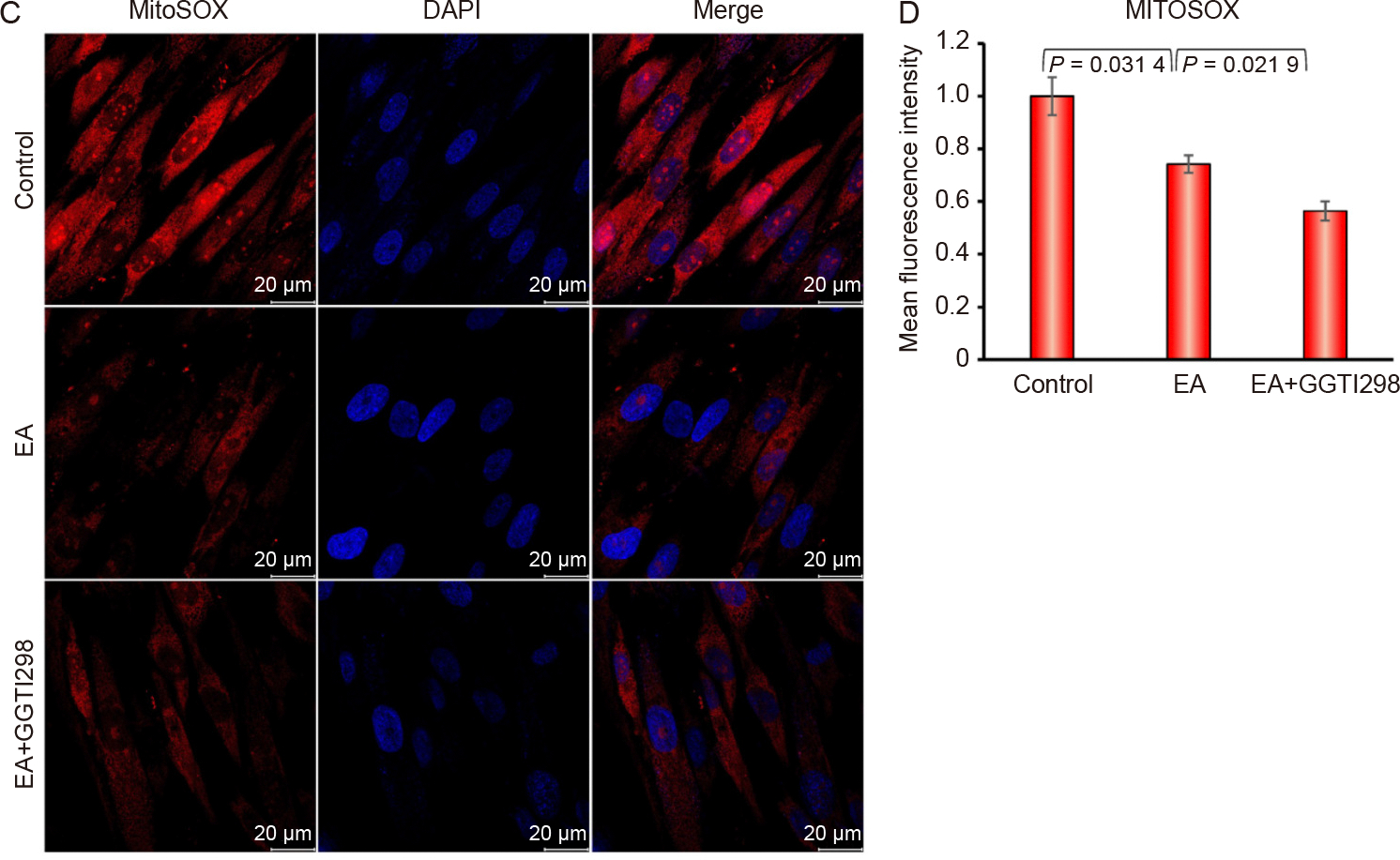
Induction of KLF2 reduced the production of intracellular and mitochondrial ROS. A. Representative confocal microscopy images of DCFDA-stained cells are shown after the induction of KLF2 using GGTI298 in the DPSCs for 7 days. B. The right panel graphically shows the quantified data. C. Representative confocal microscopy images of MitoSOX-stained cells are shown after the induction of KLF2 using GGTI298 in the DPSCs for 7 days. D. The right panel graphically shows the quantified data. Each experiment was performed at least three times, and each slide was captured in at least five different areas. For graphical data, Comparisons were conducted between con-trol and EA and between EA and GGTI298 shown as mean ±SEM from three independent experiments. Statistical signific-ance was determined using one-way ANOVA followed by Tukey’spost hoc test and the exact p-values are shown on the graph.

To evaluate the functional impact of KLF2 on cellular and mitochondrial ROS, we analyzed intracellular ROS levels via DCF-DA staining and mitochondrial superoxide levels using MitoSOX staining in DPSCs. Interestingly, intracellular ROS production was significantly reduced after the induction of KLF2 by GGTI298 compared to controls and EA-treated DPSC (Fig. 3A and 3B). Similarly, the mitochondrial ROS production was also significantly attenuated after the induction of KLF2 compared to controls and EA-treated DPSC (Fig. 3C and 3D), suggesting that KLF2 may exert a protective role against oxidative stress by reducing them.

### Effect of KLF2 on mitochondrial membrane potential and mitochondrial functions

**Fig. 4.**
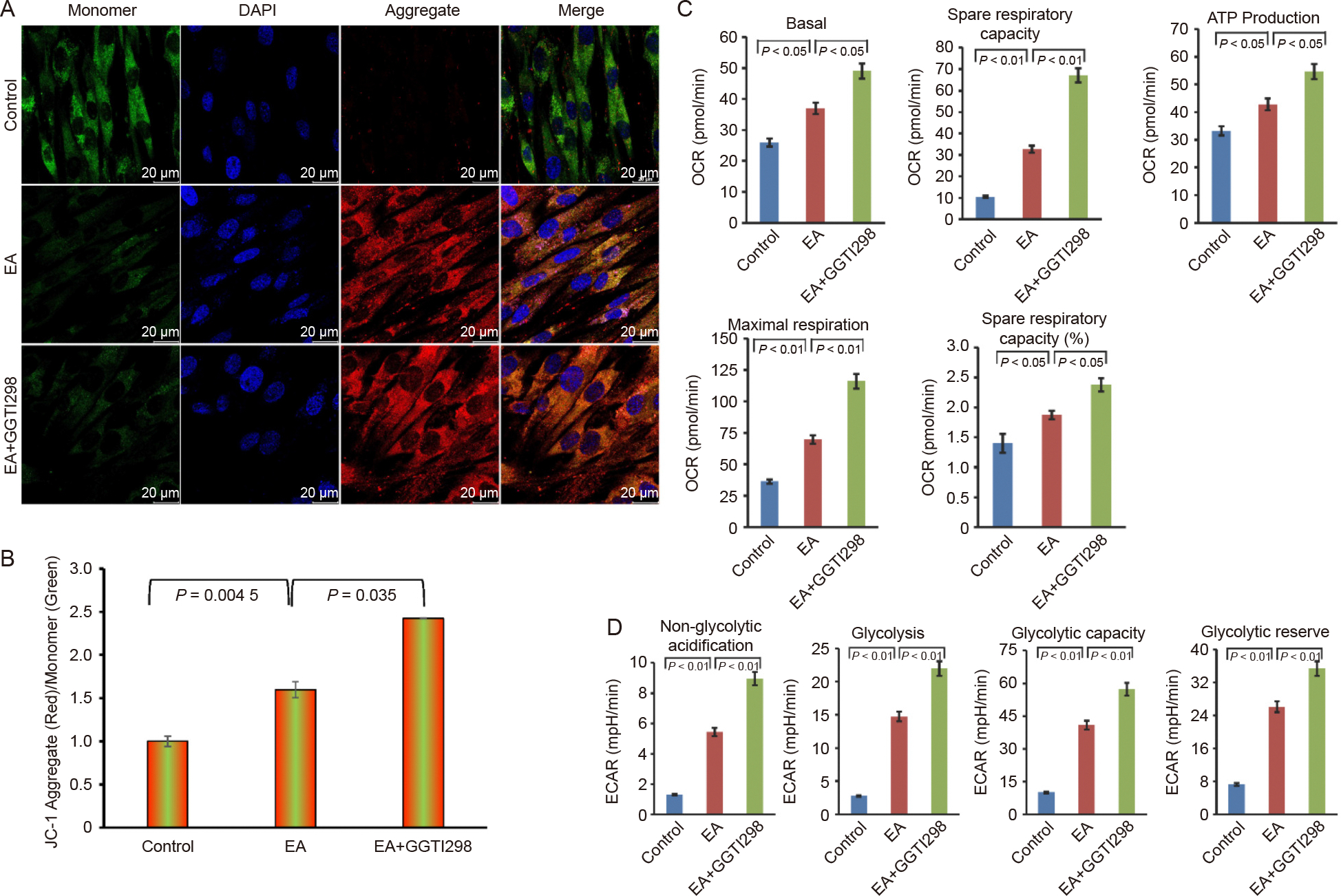
Induction of KLF2 changed the mitochondrial membrane potential and mitochondrial functions. A. Representative confocal microscopy images of JC1-stained cells are shown after the induction of KLF2 using GGTI298 in the DPSCs for 7 days. B. The bar graph represents the quantification of the mean fluorescence intensity in the aggregate/monomer ratio. C. The bar graph represents the calculated data for the stated parameters of oxygen consumption rate (OCR) in DPSCs using Seahorse XF analysis. D. The bar graph represents the calculated data for the stated parameters of extracellular acidification rate (ECAR) in DPSCs using Seahorse XF analysis. Comparisons were conducted between control and EA and between EA and GGTI298 which were shown as mean ± SEM from three independent experiments. Statistical significance was determined using one-way ANOVA followed by Tukey’s post hoc test and the exact p-values are displayed on the graph.

Then we evaluated the alterations of mitochondrial membrane potential (ΔΨm) during this KLF2-modulated EA-mediated osteogenic differentiation using JC-1 staining, where the dye is a cationic and lipophilic that forms J aggregates to give red fluorescence in the polarized mitochondria, whereas in monomeric form, depolarized mitochondria give green fluorescence. We found that after addition of EA the red fluorescence level was significantly enhanced and further increased after the induction of KLF2, whereas in control cells, JC-1 dye predominantly exhibited green fluorescence, indicating a higher proportion of depolarized mitochondria (Fig. 4A). Quantitative analysis of the red/green fluorescence ratio further confirmed that induction of KLF2 enhanced mitochondrial the membrane potential (Fig. 4B), suggesting improved mitochondrial integrity.

To further investigate how the induction of KLF2 affects mitochondrial activity, we evaluated oxygen consumption rate (OCR) in DPSCs using Seahorse XF analysis. We found that the basal respiration, spare respiratory capacity, ATP production, and maximal respiration were significantly elevated after the addition of GGTI298 to the DPSCs, indicating enhanced oxidative phosphorylation capacity (Fig. 4C, and Supplementary Fig. S3 and Fig. S4). This increase in mitochondrial respiration aligns with the high energy demand of osteogenic differentiation. Similarly, significant improvement was also observed in the levels of glycolysis capacity, glycolytic reserve, non-glycolytic acidification, and glycolysis in cells after the induction of KLF2 compared to control and EA-treated DPSCs, determined by an extracellular acidification rate (ECAR) analysis under similar experimental conditions (Fig. 4D, and Supplementary Fig. S3, Fig. S4).

### Effect of decreased KLF2 on osteogenic marker expressions

**Fig. 5.**
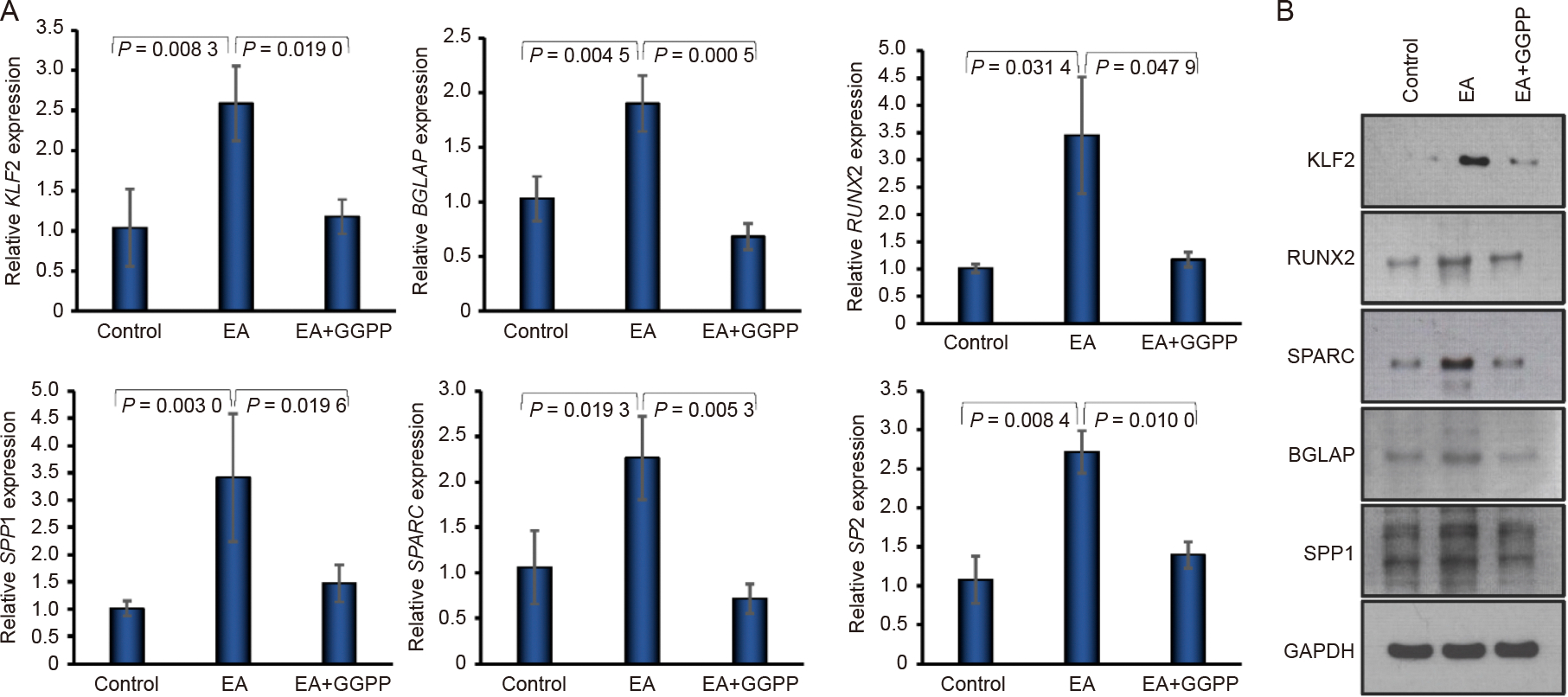
Reduction of KLF2 reduced osteogenic marker expressions. A. The mRNA expression level of KLF2, BGLAP, RUNX2, SPP1, SPARC, and SP7 was determined by qRT-PCR at 7 days of differentiation of DPSC after the addition of GGPP to the cells. B. Western blot images of KLF2, RUNX2, SPARC, BGLAP, and SPP1 at 7 days of osteoblastic differentiation using similar experimental conditions. GAPDH was used as an internal loading control. Comparisons were conducted between control and EA and between EA and GGPP which were presented as fold change and shown as mean ± SEM from three independent experiments. Statistical significance was determined using one-way ANOVA followed by Tukey’s post hoc test and the exact p-values are shown on the graph.

We next evaluated the effect of the reduction of KLF2 in DPSCs after the addition of GGPP to the DPSCs during the osteogenic differentiation for 7 days. Our RT-qPCR analysis showed that DPSCs treated with GGPP revealed a significant reduction of KLF2 expression level compared to the control or EA-added DPSCs. Similarly, the mRNA levels of several osteoblast-specific markers, including BGLAP (Osteocalcin), SP7 (Osterix), RUNX2, SPARC (Osteonectin), and SPP1 (Osteopontin), were also markedly reduced after the addition of GGPP to the DPSCs (Fig. 5A). Similarly, western blot analysis confirmed the reduced expression of the osteogenic markers, such as RUNX2, SPARC, BGLAP, and SPP1, along with KLF2, when GGPP was added to the DPSCs compared to control or EA-added DPSCs (Fig. 5 and Supplementary Fig. S4), indicating that reduction of KLF2 inhibited osteogenic differentiation-related molecules both at the mRNA and protein levels.

### Effect of decreased KLF2 on autophagy/mitophagy marker expressions

**Fig. 6.**
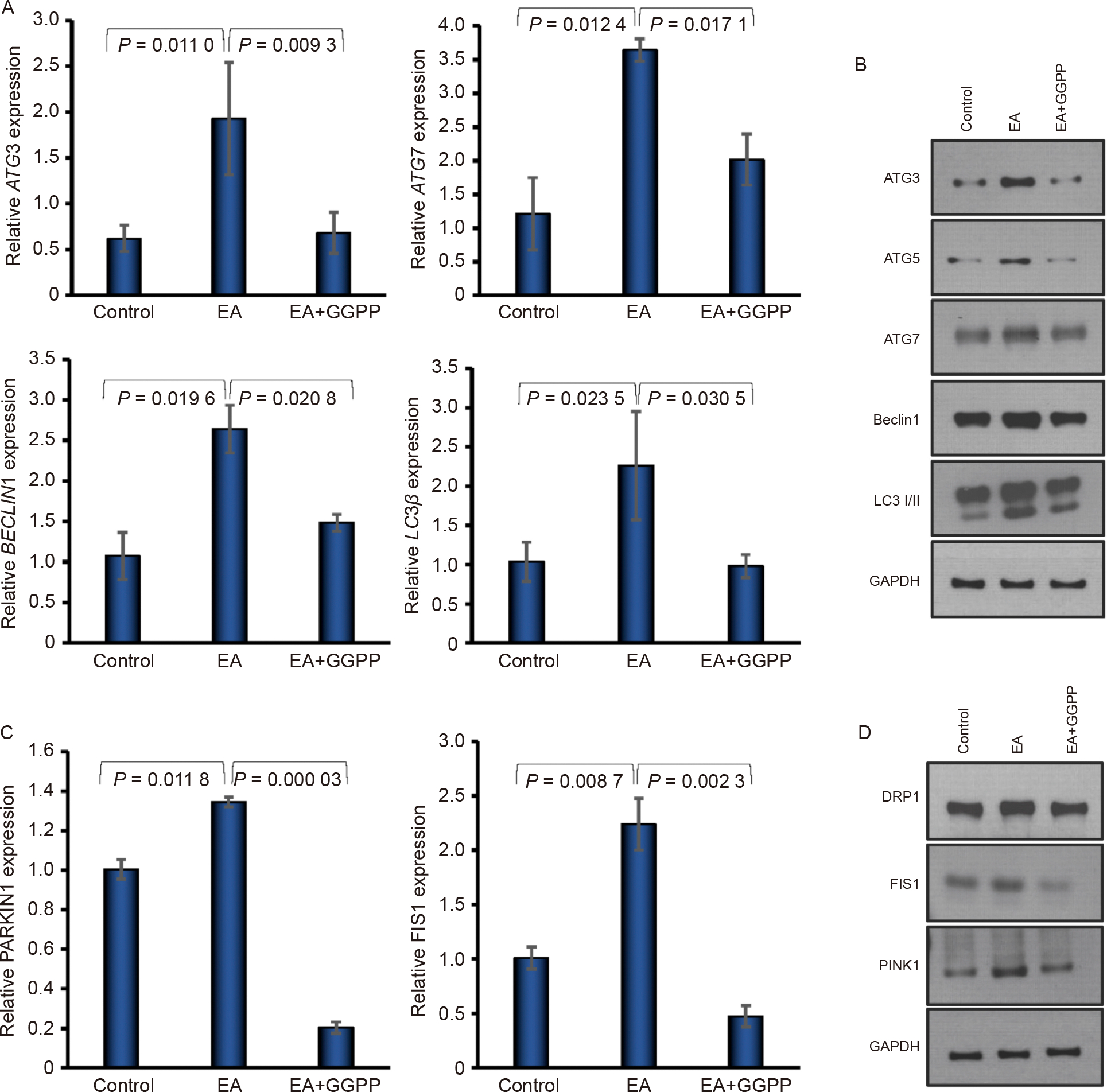
Reduction of KLF2 reduced autophagy/mitophagy marker expressions. A. Graphically shown are the qRT-PCR values of autophagy-associated markers ATG3, ATG7, BECN1, and LC3B at 7 days of differentiation of DPSC after the addition of GGPP to the cells. B. Images of western blot autophagy molecules ATG3, ATG5, ATG7, Beclin1, and LC3B were shown after 7 days of osteoblastic differentiation, keeping GAPDH as an internal loading control. C. Graphically shown are the qRT-PCR values of mitophagy-associated markers PARKIN and FIS1 at 7 days of differentiation of DPSCs after the addition of GGPP to the cells. D. Western blot images of DRP1, FIS1, and PINK1 molecules were shown after 7 days of osteoblastic differentiation, keeping GAPDH as an internal loading control. Comparisons were conducted between control and EA and between EA and GGPP which were presented as fold change and shown as mean ± SEM from three independent experiments. Statistical significance was determined using one-way ANOVA followed by Tukey’s post hoc test and the exact p-values are shown on the graph.

Next, we evaluated the effect of reduced KLF2 on autophagy-related markers. We measured the expression of key markers at the gene level using RT-qPCR. A significant decrease in the levels of autophagy-related genes, such as ATG3, ATG7, LC3B, and BECN1 after the addition of GGPP to the DPSCs compared to the control and EA-treated cells (Fig. 6A). In addition, Western blot analysis supported our findings, showing decreased protein levels of autophagy markers ATG3, ATG5, ATG7 Beclin-1, and LC3I/II, suggesting that reduction of KLF2 might lower autophagy molecules (Fig. 6B and Supplementay Fig. S5). Next, we examined whether reduced KLF2 also influenced mitophagy, which is crucial during stem cell differentiation and energy remodeling. Reduction of KLF2 led to a lower expression of mitophagy-related genes such as FIS1 and PARKIN compared to control and EA-treated cells (Fig. 6C). Similarly, at the protein level, a significant reduction in the signal intensity for DRP1, FIS1, and PINK1 was observed in KLF2-reduced DPSCs (Fig. 6D and Supplementay Fig. S5).

### Effect of decreased KLF2 on production of intracellular and mitochondrial ROS

**Fig. 7.**
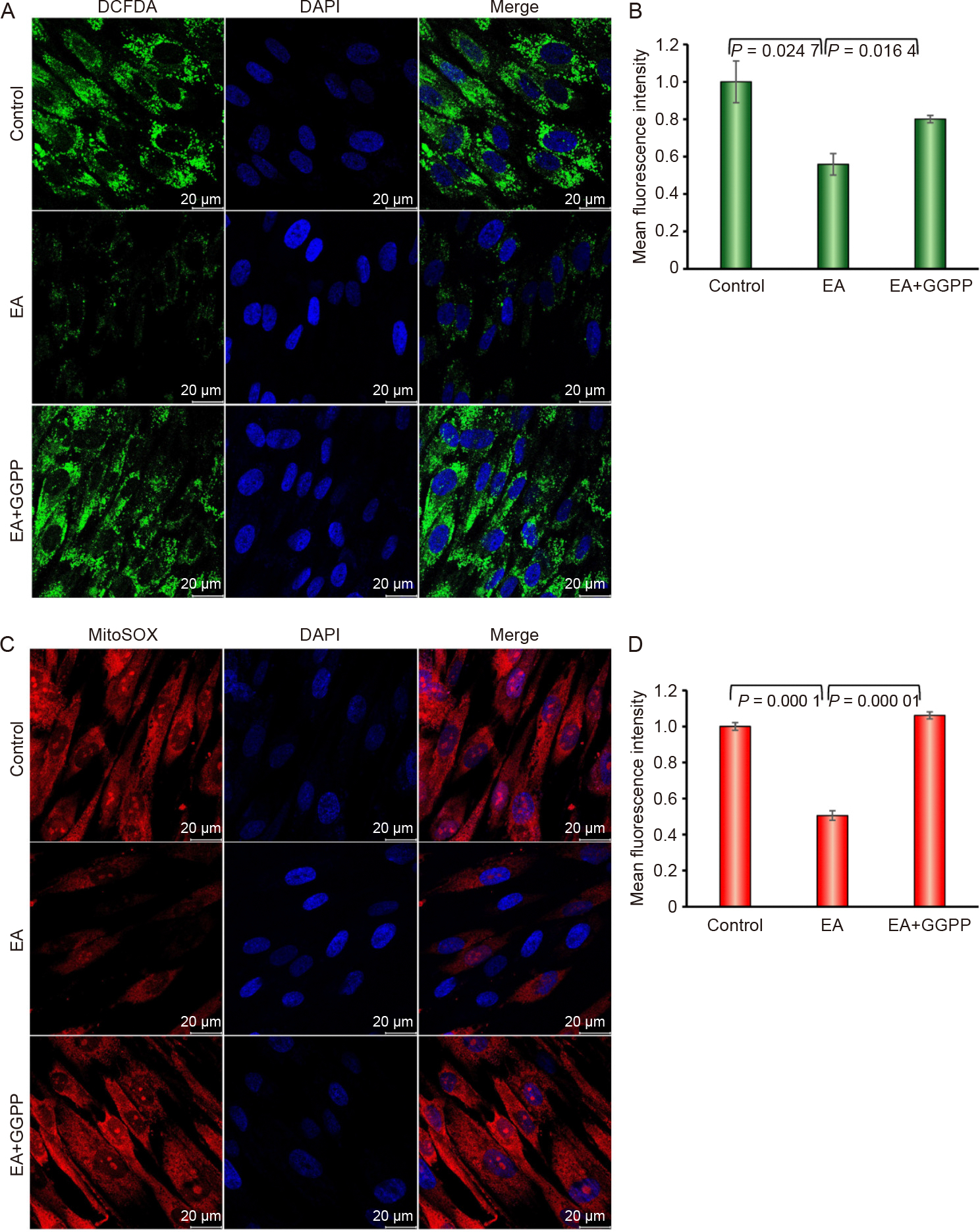
Reduction of KLF2 enhanced the production of intracellular and mitochondrial ROS. A. Representative confocal microscopy images of DCFDA-stained cells are shown after the reduction of KLF2 using GGPP in the DPSCs. B. The right panel graphically shows the quantified data. C. Representative confocal microscopy images of MitoSOX-stained cells are shown after the reduction of KLF2 using GGPP in the DPSCs for 7 days. D. The right panel graphically shows the quantified data. Each experiment was performed at least three times, and each slide was captured in at least five different areas. For graphical data, Comparisons were conducted between control and EA and between EA and GGPP shown as mean ± SEM from three independent experiments. Statistical significance was determined using one-way ANOVA followed by Tukey’s post hoc test and the exact p-values are shown on the graph.

We next assessed the functional impact on cellular and mitochondrial ROS after reduction of KLF2, by evaluating intracellular ROS levels via DCFDA and mitochondrial superoxide levels via MitoSOX staining in DPSCs. The intracellular ROS production was significantly increased after the reduction of KLF2 compared to controls and EA-treated DPSC (Fig. 7A and Fig. 7B). Similarly, the mitochondrial ROS production was also significantly enhanced after the reduction of KLF2 compared to controls and EA-treated DPSC (Fig. 7C and 7D), suggesting that KLF2 exerts a protective role against oxidative stress.

### Effect of decreased KLF2 on mitochondrial membrane potential and mitochondrial functions

**Fig. 8.**
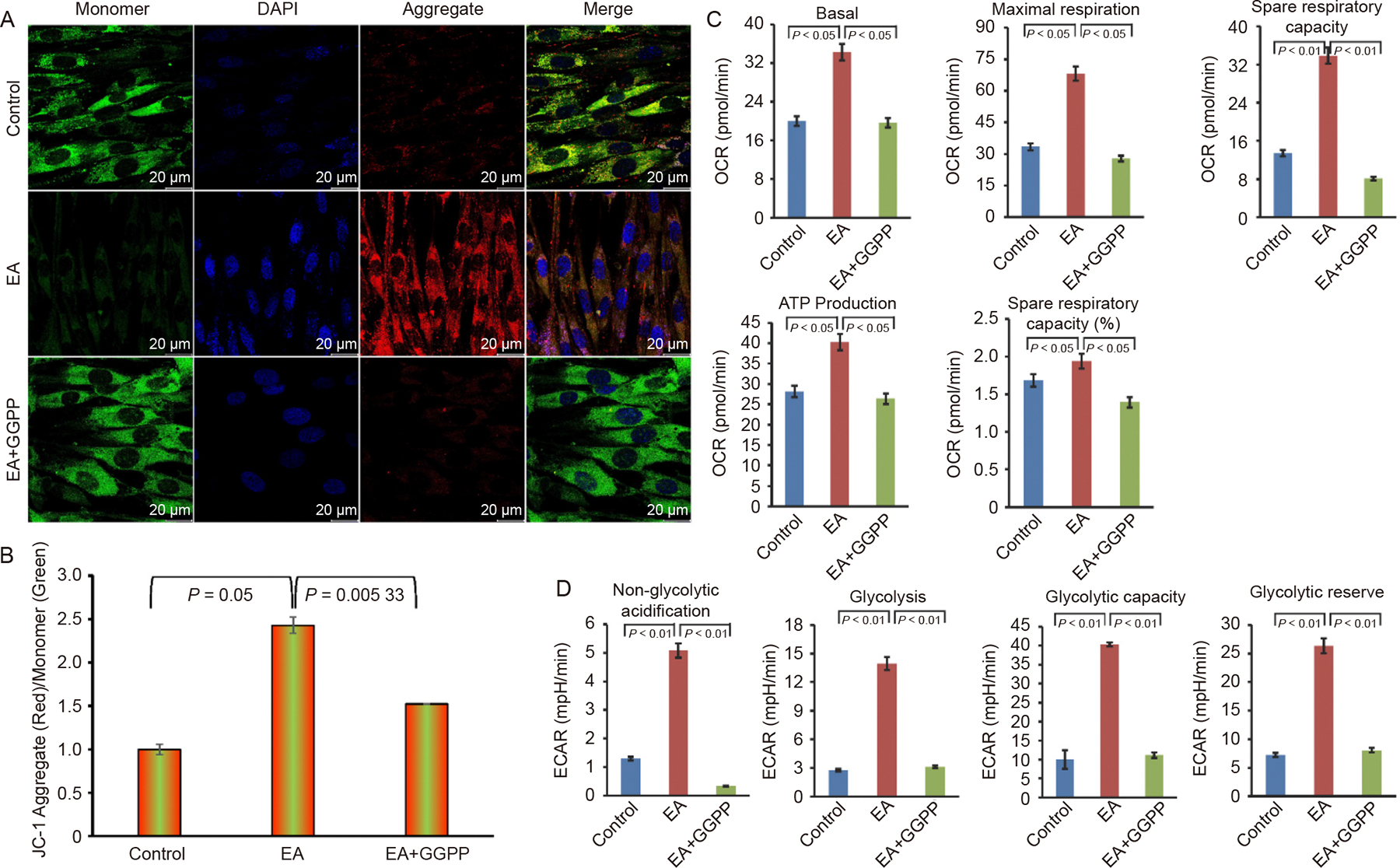
Reduction of KLF2 affected the mitochondrial membrane potential and mitochondrial functions. A. Representative confocal microscopy images of JC1-stained cells are shown after the reduction of KLF2 using GGPP in the DPSCs. B. The bar graph represents the quantification of the mean fluorescence intensity in the aggregate/monomer ratio. C. The bar graph represents the calculated data for the stated parameters of oxygen consumption rate (OCR) in DPSCs using Seahorse XF analysis. D. The bar graph represents the calculated data for the stated parameters of extracellular acidification rate (ECAR) in DPSCs using Seahorse XF analysis. Comparisons were conducted between control and EA and between EA and GGPP which were shown as mean ± SEM from three independent experiments. Statistical significance was determined using one-way ANOVA followed by Tukey’s post hoc test.

We further evaluated the alterations of mitochondrial membrane potential (ΔΨm) after the reduction of KLF2 during EA-mediated osteogenic differentiation using JC-1 staining. We observed that after the addition of EA the red fluorescence level was significantly increased and after the reduction of KLF2 the red fluorescence was significantly decreased in DPSCs, whereas in control cells, JC-1 dye predominantly exhibited green fluorescence, indicating a higher proportion of depolarized mitochondria (Fig. 8A). Quantitative analysis of the red/green fluorescence ratio further confirmed that reduction of KLF2 reduced mitochondrial the membrane potential (Fig. 8B), suggesting a compromised mitochondrial integrity.

To further investigate how the reduction of KLF2 affects mitochondrial activity, we evaluated OCR in DPSCs using Seahorse XF analysis. We found that the basal respiration, spare respiratory capacity, ATP production, and maximal respiration were significantly diminished after the addition of GGPP to the DPSCs, indicating reduced oxidative phosphorylation capacity (Fig. 8C and Supplementay Fig. S6). Similarly, significant impairment was also observed in the levels of glycolysis capacity, glycolytic reserve, non-glycolytic acidification, and glycolysis in cells after the reduction of KLF2 compared to control and EA-treated DPSCs, determined by an ECAR analysis under similar experimental conditions (Fig. 8D and Supplementay Fig. S6).

## Discussion

Osteoblast differentiation is a crucial process to maintain bone homeostasis and regeneration. Any deviations in this process lead to various skeletal disorders, including osteoporosis, osteoarthritis, and impaired fracture healing due to abnormal regeneration^[33]^. While bone marrow-derived MSC (BMMSCs) are commonly studied in this context, DPSCs have been emerging as a promising alternative due to their easy-to-collect, robust proliferative properties, and multilineage differentiation capabilities^[22,34,35]^. However, the underlying molecular mechanisms during the osteogenic differentiation of DPSCs still remain unclear^[35]^. Conversely, autophagy has been identified as a key regulator in maintaining the stemness of stem cells, initiating differentiation, and preserving mitochondrial integrity, which are crucial functions that are essential to the survival and fate of mesenchymal precursors^[36,37]^.

KLF2, a zinc-finger transcription factor that is well known for its role in many biological processes, has emerged as an important regulator in deciding the fate of MSCs^[14]^. It modulates osteogenesis by transcriptionally activating RUNX2, a master regulator of osteoblast differentiation and repressing adipogenic transcription factors such as PPARγ and C/EBPα, thereby favoring osteogenic lineage commitment over adipogenesis^[14,38]^.

Several studies showed that there is a functional cross-talk between autophagy, mitophagy, and the transcription factor KLF2, a well-established essential cellular mechanism involved in maintaining intracellular homeostasis, particularly during highenergy processes like osteogenic differentiation. Several studies demonstrate that KLF2 shares regulatory overlap with autophagic machinery^[39]^. For instance, statins, a class of lipid-lowering agents that have been shown to upregulate both KLF2 and autophagy in prostate cancer cells^[40]^. Moreover, regulatory pathways involving KLF and autophagy have been linked to aging and vascular dysfunction across different species^[41]^. These observations indicate that KLF2 could act as a potential upstream regulator of autophagy, though its precise role in osteogenesis, particularly in DPSC lineage commitment, has yet to be fully understood.

In the present study, we aimed to investigate the role of KLF2 in EA-mediated osteogenic differentiation of DPSCs, with a focus on autophagy, mitophagy, and mitochondrial dynamics. From our previous findings, we demonstrated that EA significantly upregulates KLF2 expression in DPSCs undergoing osteogenic induction^[31]^. Additionally, we found that the addition of EA to the DPSCs led to an increase in the expression of osteogenic markers such as RUNX2, SP7, SPARC, and BGLAP, supporting previous studies on its bone-forming effects. Further in our current study, we showed that the induction of KLF2 further enhanced this effect, indicating a possible synergistic role of EA and KLF2 in promoting OB differentiation of DPSCs. However, during our initial experiments, treatment with GGTI298 or GGPP alone did not show any change in KLF2 expression or osteogenic marker expression levels in DPSCs (data not shown), and therefore these groups were not pursued further. These groups showed effects only in the presence of EA, indicating a possible interaction-dependent role of KLF2 signaling in EA-mediated osteogenic differentiation.

In addition to osteogenic enhancement, induction of KLF2 by the GGTI298 and EA treatment led to a significant increase in autophagy-associated genes such as ATG3, ATG5, BECN1, and LC3B, indicating enhanced autophagy-related signaling during OB differentiation of DPSCs. Our results are consistent with earlier findings showing that autophagy supports osteoblast maturation by facilitating matrix mineralization and maintaining intracellular energy balance^[26,42]^. Similarly, our results are also consistent with previous studies that showed a connection between KLF2 expression and increased osteogenesis as well as autophagic activity in mesenchymal stem cells^[37]^. While EA and KLF2 modulation increased LC3-II and Beclin-1 levels, we understand that, without autophagic flux assays using lysosomal inhibitors (e.g., Bafilomycin A1), these changes may reflect either enhanced autophagosome formation or impaired autophagosome clearance. Furthermore, we also observed an increase in mitochondrial fission-related proteins such as DRP1, FIS1, and mitophagy-associated protein PINK1, along with increased mitochondrial membrane potential and reduced ROS, indicating enhanced mitochondrial dynamics and function. While we observed changes in FIS1, DRP1, and PINK1 levels, these are associated with mitochondrial dynamics, but these proteins are not definitive markers of mitophagy and hence require further studies. Similar results were also reported when mouse skin-derived MSCs were differentiated into osteocytes, chondrocytes, and adipocytes^[43,44]^. These findings emphasize the complex role of KLF2 in maintaining mitochondrial homeostasis during OB differentiation and suggest a coordinated regulatory role of KLF2 in managing mitochondrial quality control and osteoblast differentiation. Moreover, these results indicate that KLF2 works in synergy with EA to promote a pro-osteogenic intracellular environment, probably by maintaining mitochondrial integrity and balancing the energy that is essential for the differentiation of osteoblasts from DPSCs.

In contrast, to further confirm the function of KLF2’s involvement in these processes, we reduce the expression of KLF2 with GGPP, which has been reported to reduce statin-triggered KLF2 expression and hinder autophagic activities, greatly reducing the osteogenic impacts of EA^[45]^. The reduction of KLF2 led to a decrease in the expression of all the studied osteogenic and autophagic markers during EA-mediated osteogenic differentiation, indicating that KLF2 could be an essential mediator for the differentiation effects triggered by EA. These findings support earlier research indicating that the knockdown of KLF2 resulted in reduced autophagy and hindered differentiation in various cell models^[37,46]^.

Additionally, we found that the reduction of KLF2 led to an increase in the levels of intracellular and mitochondrial ROS during EA-mediated osteogenic differentiation, indicating that KLF2 could be an essential mediator for mitochondrial quality and functions. It was reported that the impaired mitophagy signaling and increased intracellular and mitochondrial ROS levels in KLF2-deficient cells further emphasize its importance in maintaining mitochondrial quality control during EA-mediated differentiation^[47]^. The observed decline in autophagic flux and mitophagic clearance in the absence of KLF2 supports the idea that KLF2 acts as an upstream transcriptional regulator of these pathways, as shown in models related to the vascular and neuronal system^[48,49]^.

Other than its function in osteogenic differentiation, KLF2 acts as a key transcriptional regulator that combines responses to oxidative stress, maintenance of mitochondrial balance, and cellular metabolism. Several prior studies have shown that KLF2 functionally interacts with nuclear factor erythroid 2-related factor 2 (NRF2), which is a principal regulator of antioxidant defense. This enhances the transcription of redox-protective genes like heme oxygenase-1 (HO-1) and NAD(P)H quinone oxidoreductase-1 (NQO1), thus preserving cellular redox balance during stressful situations^[50,51]^. Likewise, FOXO3a exhibits overlapping regulatory roles with KLF2 in regulating autophagy, mitophagy, and the turnover of mitochondria, all of which are essential for maintaining and differentiating stem cells^[52,53]^. The KLF2 activation mediated by EA observed in this study might therefore work together with transcriptional programs dependent on NRF2 and FOXO3a to enhance antioxidant capacity, manage mitochondrial quality, and support osteogenic commitment in DPSC. This potential interaction indicates a wider regulatory network where KLF2 orchestrates redox and metabolic homeostasis alongside other transcription factors responsive to stress, thereby contributing to increased robustness and osteogenic capability in stem cells.

Studies have reported that GGTI298 acts by inhibiting geranylgeranyltransferase-I, which could affect multiple geranylgeranylated proteins, such as several Rho GTPases, that could lead to possible off-target effects on other prenylation-dependent proteins and pathways that produce effects other than direct modulation of KLF2^[54,55]^. Similarly, GGPP restores geranylgeranylation could alter protein prenylation and can rescue phenotypes caused by prenylation blockade and also influence pathways beyond KLF2 regulation^[56–58]^. These findings are consistent with prior work showing that disruptions of the mevalonate RhoA pathway can alter KLF2 expression^[59,60]^.

Although our data show that KLF2 mediates EA-induced differentiation, we did not perform any rescue experiments. To determine whether restoring KLF2 levels is sufficient to counteract the inhibitory effects of GGPP, we can overexpress KLF2 in GGPP-treated DPSCs, which could help confirm the functional requirement of KLF2 in EA-mediated osteogenic differentiation. Including such gain-of-function studies could provide stronger mechanistic clarity and further validate the central role of KLF2 in coordinating the EA-mediated osteogenic response.

Although the present study elucidates a potential mechanistic role of KLF2 during EA-mediated osteogenic differentiation, certain limitations still need to be addressed. Firstly, modulating KLF2 expression using pharmacological agents such as GGTI298 and GGPP acts indirectly and may show off-target effects on other prenylation-dependent signaling proteins. Although these compounds effectively altered KLF2 expression and associated osteogenic responses, their off-target effects still need further investigation. In order to strengthen the specificity, future studies employing genetic modulation approaches, such as KLF2 overexpression or CRISPR-mediated knockdown, would provide more direct evidence for the specific role of KLF2 in EA-mediated osteogenesis and eliminate potential off-target effects. Additionally, while our findings in DPSC provide strong in vitro support, they still require in vivo validation using a bone regeneration or defect repair model to confirm the physiological relevance of the EA-KLF2-autophagy/mitophagy axis under regenerative conditions.

To confirm the pathway of autophagy regulation in DPSCs, we will need to perform autophagic flux assays using lysosomal inhibitors (e.g., Bafilomycin A1, Chloroquine) and live-cell autophagosome/lysosome tracking to distinguish between increased initiation of autophagy versus impaired degradation. Additionally, we will perform LC3-mitochondria colocalization or Parkin translocation assays to directly confirm mitophagic activity in this experimental system. Also, functional studies involving DRP1 inhibition or genetic knockdown of PINK1/Parkin could clarify whether mitochondrial remodeling is necessary for KLF2mediated osteogenesis. In order to strengthen the ROS dynamics, flow cytometry-based validation would offer additional singlecell resolution; however, this was not performed in the current study, and we plan to incorporate that in the future to further strengthen the quantitative evaluation of ROS dynamics in DPSCs.

## Conclusion

Overall, these results indicate that EA promotes osteogenic differentiation of DPSCs by upregulating KLF2, which in turn plays a role in regulating autophagy and mitophagy to facilitate cellular differentiation and survival. The activation of autophagy may be essential for degrading and recycling damaged organelles and macromolecules during lineage commitment, whereas mitophagy ensures mitochondrial health, a significant factor in determining the fate of stem cells. Our findings provide a novel perspective on the relationship between KLF2 and autophagy in bone biology and suggest that modulation of KLF2 activity may improve the efficacy of EA or similar polyphenolic compounds for regenerative applications. From a translational perspective, these findings have considerably important implications. Induction of KLF2 or increasing its activity could act as a promising therapeutic approach to enhance bone regeneration, especially in conditions marked by impaired osteogenesis, such as osteoporosis or age-related bone loss. Additionally, KLF2-targeted therapies may enhance the efficacy of stem cell-based approaches by promoting a more favorable metabolic and epigenetic landscape for differentiation.

### Ethical approval and consent to participate

This study did not require approval from an institutional ethics committee, as it involved retrospective analysis of anonymized patient data collected as part of standard clinical care. No interventions outside standard practice were conducted, and no identifiable personal information was used. Therefore, patient consent to participate was not required.

## Supplementary Material

Supplementary

## Abbreviations

ANOVA: Analysis of Variance
ATG: Autophagy-related protein
ATP: Adenosine triphosphate
BECN1: Beclin-1
BGLAP: Osteocalcin
BMMSCs: Bone marrow-derived MSC
BMPs: Bone morphogenetic proteins
BSA: Bovine serum albumin
C/EBPα: CCAAT/enhancer-binding protein alpha
cDNA: Complementary deoxyribonucleic acid
CO_2_: Carbon dioxide
Cq: Quantification cycle
DCFDA: 2’,7’-dichlorodihydrofluorescein diacetate
DMEM: Dulbecco’s Modified Eagle’s Medium
DMSO: Dimethyl sulfoxide
DPSCs: Dental pulp-derived stem cells
DRP1: Dynamin-Related Protein 1
EA: Ellagic acid
ECAR: Extracellular acidification rate
FBS: Fetal bovine serum
FCCP: Carbonyl cyanide 4-(trifluoromethoxy) phenylhydrazone
FIS1: Mitochondrial Fission Protein 1
FOXO3a: Forkhead box O3a Rho
GAPDH: Glyceraldehyde 3-phosphate dehydrogenase
GGPP: Geranylgeranyl pyrophosphate
GGTI298: Geranylgeranyl transferase inhibitor 298
GTPase: Rho guanosine triphosphatases
HBSS: Hanks’ Balanced Salt Solution
HO-1: Heme oxygenase-1
HRP: Horse radish peroxidase
KLF2: Krüppel-like factor 2
LC3B: Microtubule-associated protein 1 light chain 3 beta
MEM-a: Minimun essential medium a
MSCs: Mesenchymal stem cells
NIH: National Institutes of Health
NQO1: NAD(P)H quinone oxidoreductase-1
NRF2: Nuclear factor erythroid 2-related factor 2
OCR: Oxygen consumption rate
PARKIN: E3 ubiquitin protein ligase
PBS: Phosphate-buffered saline
PINK1: PTEN-induced putative kinase 1
PPARγ: Peroxisome proliferator-activated receptor gamma
RANKL: Nuclear factor kappa-B ligand
RIPA: Radioimmunoprecipitation Assay
RNA: Ribonucleic acid
ROS: Reactive oxygen species
RT-qP-CR: Reverse transcriptase quantitative polymerase chain reaction
RUNX2: Runt-related transcription factor 2
SDS-PAGE: Sodium Dodecyl Sulfate Polyacrylamide Gel Electrophoresis
SEM: Standard error of mean
SP7: Osterix
SPARC: Osteonectin
SPP1: Osteopontin
TBST: Tris-buffered saline with Tween-20
