# Supplementary material for: KLF2 Promotes Ellagic Acid-Mediated Osteogenic Differentiation of Dental Pulp-Derived Stem Cells via Autophagy and Mitochondrial Regulation
Source: Dis Res. Author manuscript; Available in PMC 2026 Apr 22. (PMC13099239; doi:10.54457/dr.202504006)
Supplement: Supplementary [file NIHMS2153826-supplement-Supplementary.pdf]

Supplementary

KLF2 Promotes Ellagic Acid-Mediated Osteogenic Differentiation of Dental Pulp-Derived Stem Cells via Autophagy and Mitochondrial Regulation

Prathyusha Naidu[1], Md Sariful Islam Howlader[1], Surajit Hansda[1], Manjusri Das[1], and Hiranmoy Das[1,#]

[1]Department of Pharmaceutical Sciences, Jerry H. Hodge School of Pharmacy, Texas Tech University Health Sciences Center, Amarillo, Texas 79106, USA.

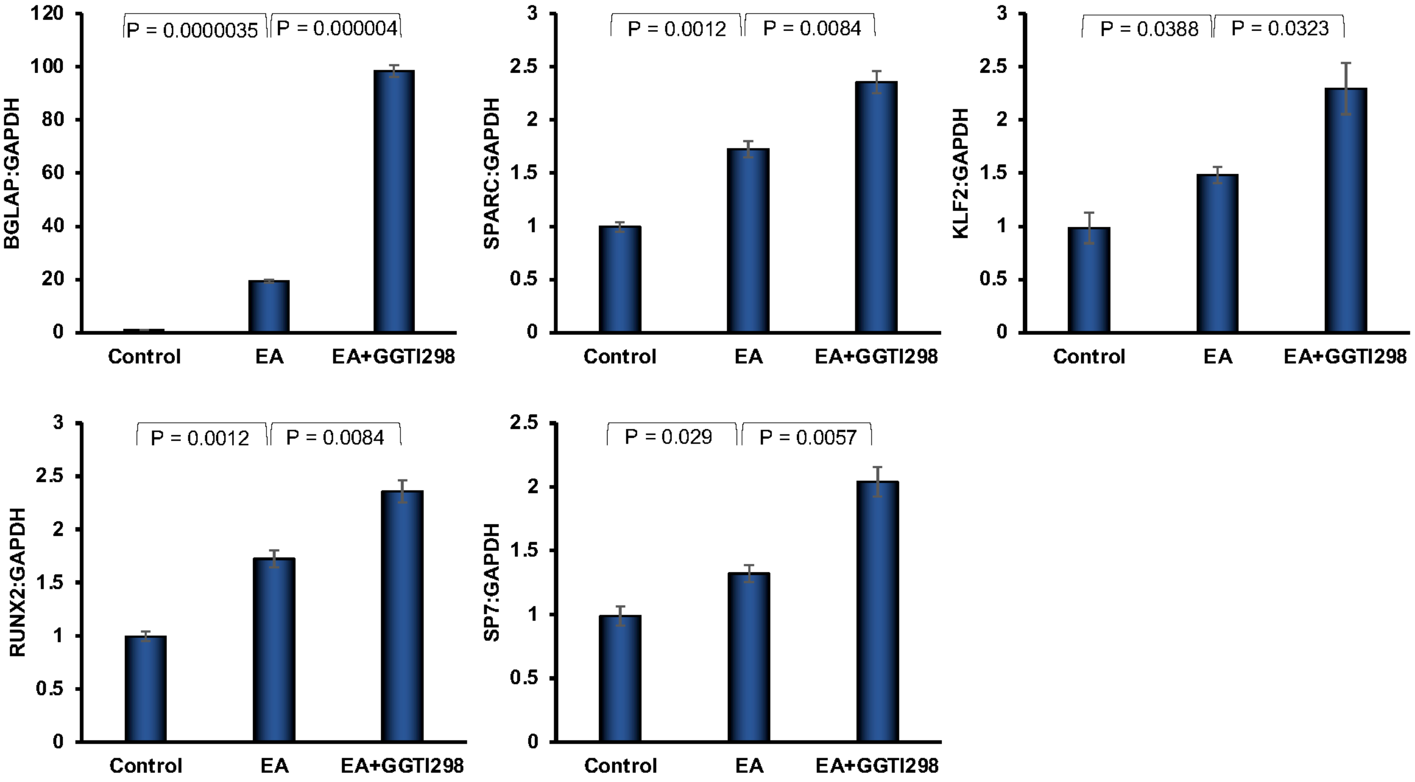

Figure S1. Bar graphs show the quantified level of BGLAP, SPARC, KLF2, RUNX2, and SP7 proteins with respect to GAPDH protein, as shown in the original Figure 1B.

Supplementary Figure 1

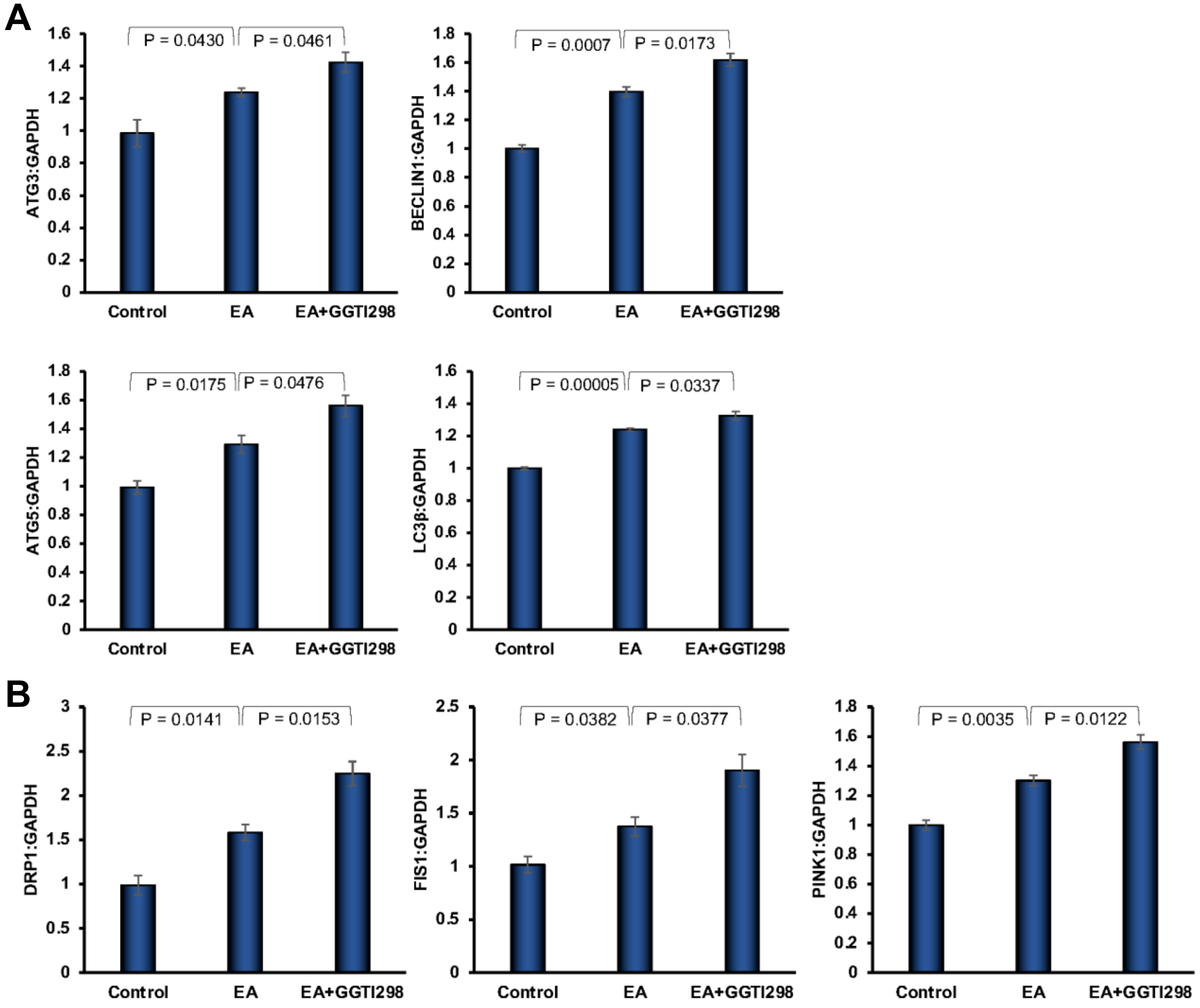

Figure S2. A. Bar graphs show the quantified level of ATG3, ATG5, Beclin1, and LC3B proteins compared to GAPDH protein, as shown in the original Figure 2B. **B**. Bar graphs show the quantified level of DRP1, FIS1, and PINK1 proteins, compared to the GAPDH level, as shown in the original Figure 2D.

Supplementary Figure 2

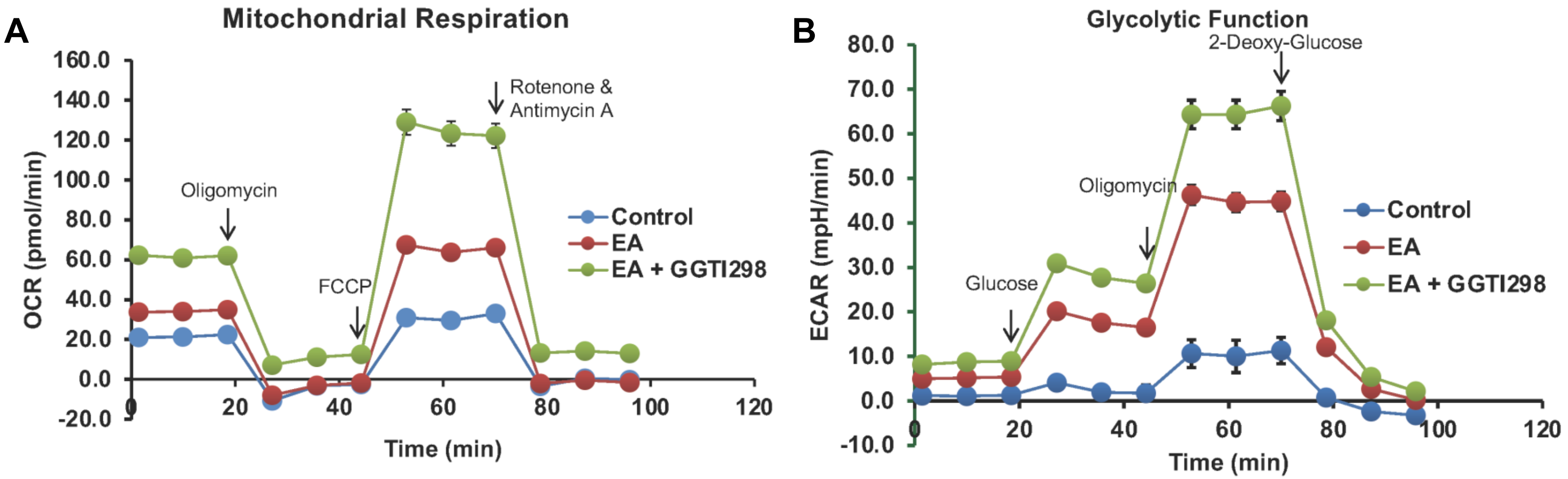

Figure S3. A. Line graphs showing the original seahorse extracellular flux analysis data of oxygen consumption rate (OCR) in cells after osteoblastic differentiation of dental pulp-derived stem cells (DPSC) in the presence of GGTI298. **B**. Line graphs showing the original Seahorse flux analysis data of extracellular acidification rate (ECAR) in cells after osteoblastic differentiation of dental pulp-derived stem cells (DPSC) in the presence of GGTI298.

Supplementary Figure 3

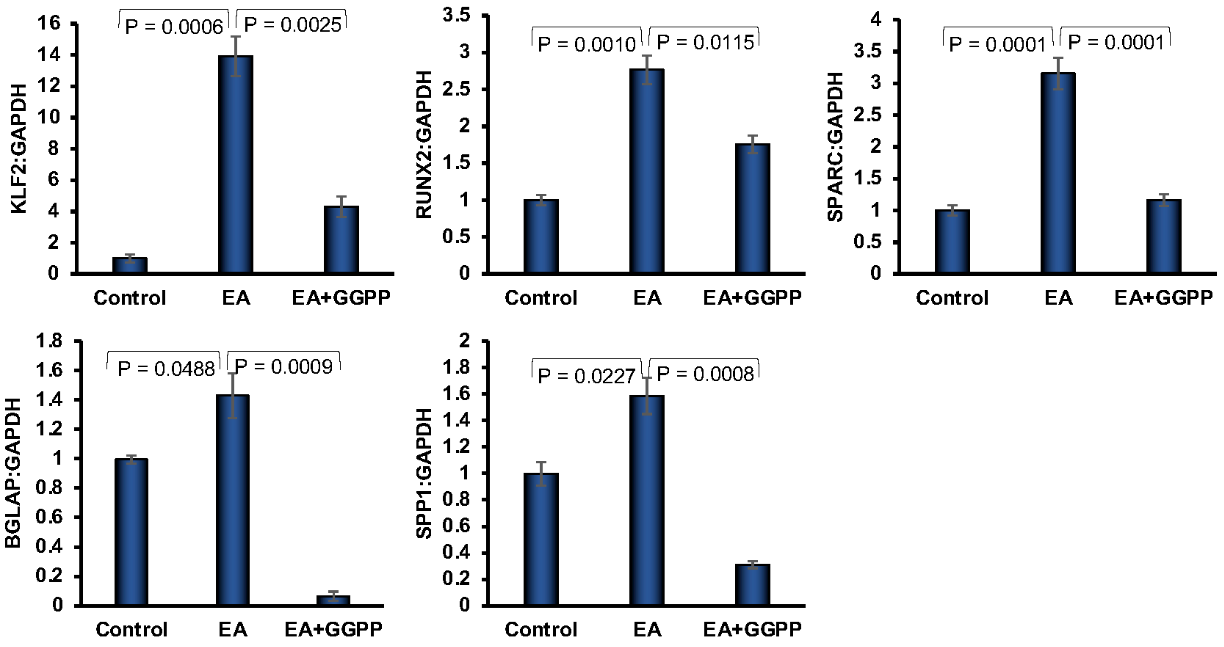

Figure S4. Bar graphs show the quantified level of KLF2, RUNX2, SPARC, BGLAP, and SPP1 proteins with respect to GAPDH protein, as shown in the original Figure 5B.

Supplementary Figure 4

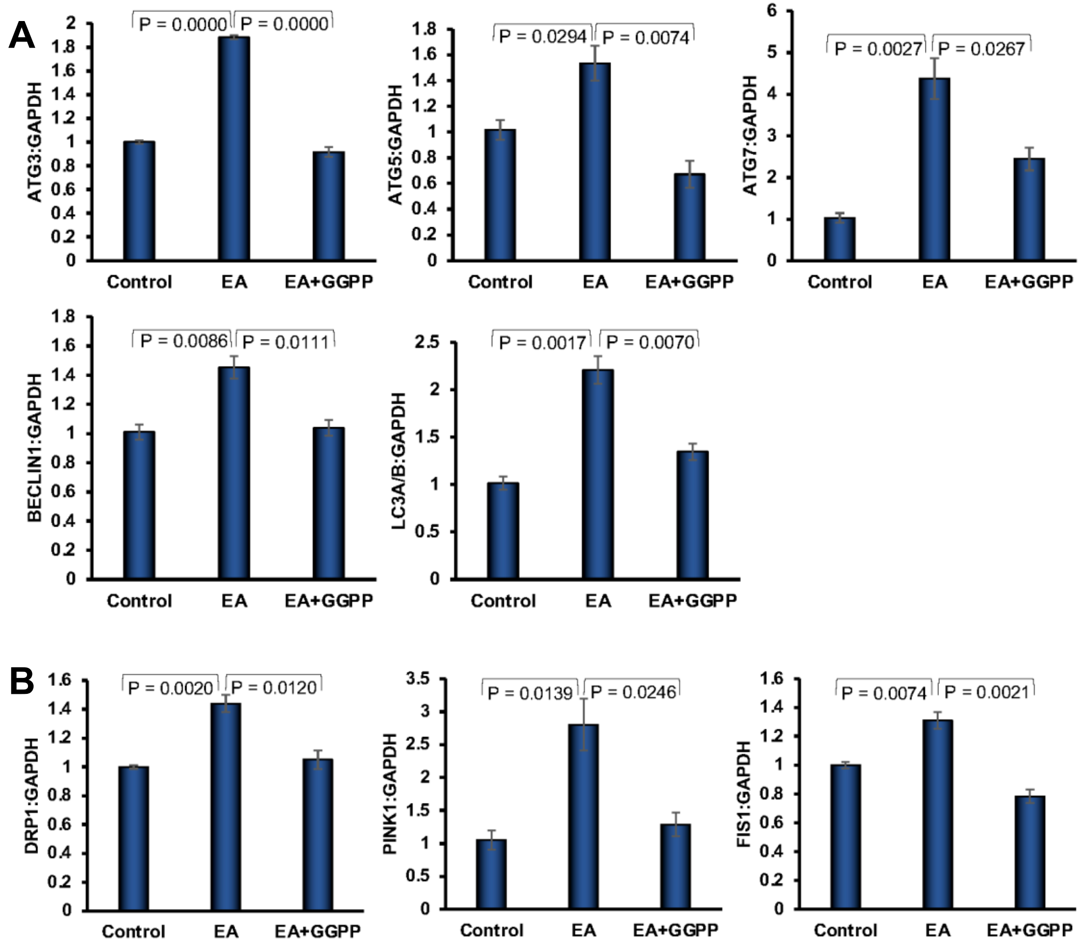

Figure S5. A. Bar graphs show the quantified level of ATG3, ATG5, ATG7, Beclin1, and LC3B proteins compared to GAPDH protein, as shown in the original Figure 6B. **B**. Bar graphs show the quantified level of DRP1, PINK1, and FIS1 proteins, compared to the GAPDH level, as shown in the original Figure 6D.

Supplementary Figure 5

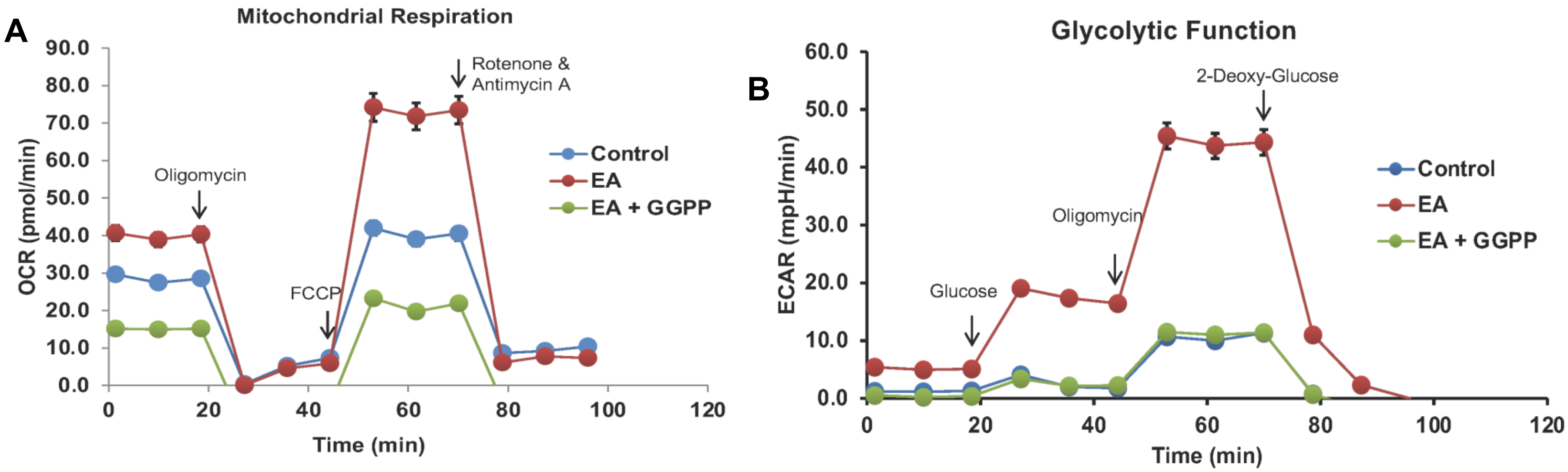

Figure S6. A. Line graphs showing the original seahorse extracellular flux analysis data of oxygen consumption rate (OCR) in cells after osteoblastic differentiation of dental pulp-derived stem cells (DPSC) in the presence of GGPP. **B**. Line graphs showing the original Seahorse flux analysis data of extracellular acidification rate (ECAR) in cells after osteoblastic differentiation of dental pulp-derived stem cells (DPSC) in the presence of GGPP.

Supplementary Figure 6

Table S1. Primer sequences of genes used in quantitative RT-PCR experiments.

**1**	**ATG3 Forward**	**5'-GTG ATG GCG GAT GGG TAG AT-3'**
**2**	ATG3 Reverse	5'-CCT TGT ATC TAG GGT AGC CTC A-3'
**3**	ATG5 Forward	5'-ACA AGC AAC TCT GGA TGG GA-3'
**4**	ATG5 Reverse	5'-GCC AAG CTT GTA ACC AGG GA-3'
**5**	ATG7 Forward	5'-GCA AGA GAA AGC TGG TCA TCA-3'
**6**	ATG7 Reverse	5'-GCC AAG CTT GTA ACC AGG GA-3'
**7**	KLF2 Forward	5'-ACA CAG GTG AGA AGC CCT AC-3'
**8**	KLF2 Reverse	5'-ATGTGCCGTTTCATGTGCA-3'
**9**	RUNX2 Forward	5'-CGC CTC ACA AAC AAC CAC AG-3'
**10**	RUNX2 Reverse	5'-GCT GTT TGA TGC CAT AGT CCC-3'
**11**	SP7 Forward	5'- CAT TCT GGG CTT GGG TAT CT -3'
**12**	SP7 Reverse	5'- GGC CTG AGA TGA GAG TTT GT -3'
**13**	SPP1 Forward	5'- CGA GGA GTT GAA TGG TGC ATA -3'
**14**	SPP1 Reverse	5'- TCC AGC TGA CTC GTT TCA TAA C -3'
**15**	BECN1 Forward	5'- GCT GCC GTT ATA CTG TTC TGG-3'
**16**	BECN1 Reverse	5'- TCT CCA CAT CCA TCC TGT AGG-3'
**17**	PARKIN1 Forward	5'- GTG TTT GTC AGG TTC AAC TCC A-3'
**18**	PARKIN1 Reverse	5'- GAA AAT CAC ACG CAA CTG GTC-3'
**19**	DRP1 Forward	5'- AGT CAT GGA GGC GCG GCT AAT TC-3'
**20**	DRP1 Reverse	5'- CGG TCC CAC TAC GAC GAT TT-3'
**21**	FIS1 Forward	5'- GAA CTA CCG GCT CAA GGA ATA C-3'
**22**	FIS1 Reverse	5'- CCC ACG AGT CCA TCT TTC TTC -3'
**23**	β-ACTIN Forward	5'-CAC CAA CTG GGA CGA CAT-3'
**24**	β-ACTIN Reverse	5'-ACA GCC TGG ATA GCA ACG-3'

Supplementary Table S1

Table S2. Details of antibodies used in this study.

S. No.	Antibody and their Catalogue No.	Company	Dilutions for Western Blot
1	SPARC(8725 S)	Cell Signaling	1:1000
2	BGLAP(AB93876)	Abcam	1:100
3	SPP1(ab8448)	Abcam	1:1000
4	SP7(ab94744)	Abcam	1:2000
5	RUNX2(8486 S)	Cell Signaling	1:1000
6	KLF2 (ab236507)	Abcam	1:1000
7	Beclin1 (3738 S)	Cell Signaling	1:1000
8	ATG3 (3415 S)	Cell Signaling	1:1000
9	ATG7 (8558 S)	Cell Signaling	1:1000
10	LC3B (12741S)	Cell Signaling	1:1000
11	PARKIN (2132S)	Cell Signaling	1:1000
12	DRP1 (ab184247)	Abcam	1:1000
13	FIS1 (1095S)	Cell Signaling	1:1000
14	PINK1(6946 S)	Cell Signaling	1:1000
15	GAPDH(2118 S)	Cell Signaling	1:1000
16	Anti-rabbit IgG, HRP-linked Antibody (7074)	Cell Signaling	1:3000
17	Anti-mouse IgG, HRP-linked Antibody (7076)	Cell Signaling	1:3000
18	ATG5 ()	Cell Signaling	N/A

Supplementary Table S2
